# Identification of suitable habitats and priority conservation areas under climate change scenarios for the Chinese alligator (*Alligator sinensis*)

**DOI:** 10.1002/ece3.11477

**Published:** 2024-05-30

**Authors:** Liuyang Yang, Jiangnan Ling, Lilei Lu, Dongsheng Zang, Yunzhen Zhu, Song Zhang, Yongkang Zhou, Pingsi Yi, En Li, Tao Pan, Xiaobing Wu

**Affiliations:** ^1^ Life Sciences Anhui Normal University Wuhu Anhui China; ^2^ The Anhui Provincial Key Laboratory of Biodiversity Conservation and Ecological Security in the Yangtze River Basin Anhui Normal University Wuhu Anhui China; ^3^ National Long‐term Scientific Research Base for Chinese Alligator Artificial Breeding and Protection in Anhui Anhui Research Center for Chinese Alligator Reproduction Xuancheng Anhui China

**Keywords:** climate change, distribution, habitat suitability, priority conservation areas, the Chinese alligator

## Abstract

Amphibians and reptiles, especially the critically endangered Chinese alligators, are vulnerable to climate change. Historically, the decline in suitable habitats and fragmentation has restricted the distribution of Chinese alligators to a small area in southeast Anhui Province in China. However, the effects of climate change on range‐restricted Chinese alligator habitats are largely unknown. We aimed to predict current and future (2050s and 2070s) Chinese alligator distribution and identify priority conservation areas under climate change. We employed species distribution models, barycenter migration analyses, and the Marxian model to assess current and future Chinese alligator distribution and identify priority conservation areas under climate change. The results showed that the lowest temperature and rainfall seasonality in the coldest month were the two most important factors affecting the distribution of Chinese alligators. Future predictions indicate a reduction (3.39%–98.41%) in suitable habitats and a westward shift in their distribution. Further, the study emphasizes that suitable habitats for Chinese alligators are threatened by climate change. Despite the impact of the Anhui Chinese Alligator National Nature Reserve, protection gaps persist, with 78.27% of the area lacking priority protected area. Our study provides crucial data for Chinese alligator adaptation to climate change and underscores the need for improved conservation strategies. Future research should refine conservation efforts, consider individual plasticity, and address identified limitations to enhance the resilience of Chinese alligator populations in the face of ongoing climate change.

## Graphical abstract

This study aims to assess the threat to the habitats of Chinese alligators under climate change by utilizing species distribution models and barycenter migration analyses. In addition, this study combined species distribution models and systematic conservation planning tools to identify priority protection areas for Chinese alligators under climate change and provide data support for the protection of Chinese alligators under climate change.

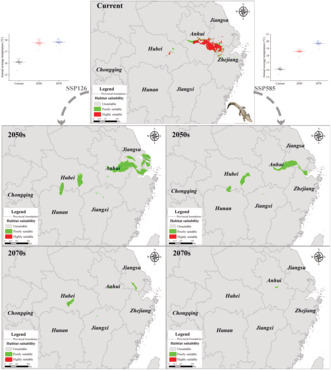

## 1 INTRODUCTION

Climate change is one of the greatest challenges facing global biodiversity in the 21st century (IPCC, 2001a, 2001b). According to the Intergovernmental Panel on Climate Change (IPCC), surface temperatures have already increased by at least 1.5°C in the last century (Gillett et al., 2021; IPCC, 2023). This temperature rise is gradually altering the functions of global ecosystems and biodiversity patterns (Prakash, 2021). Changes in the suitability of existing habitats represent a major challenge associated with climate change (Littlefield et al., 2019). Since a suitable habitat is crucial for species survival and reproduction (Brown, 1964; Krausman, 1999), accurate evaluation of the species habitat quality becomes imperative.

Herpetofauna face a greater risk from climate change than other vertebrate groups (Bickford et al., 2010; Newbold, 2018), which is mainly due to their physiological constraints and close association with the environment. Climate change has altered the phenology and distribution of herpetofauna (Gibbs & Breisch, 2001; Li et al., 2013). For instance, it can cause considerable changes in the spatial distribution of amphibian diversity (Diele‐Viegas et al., 2020; Duan et al., 2016), alter population structures (Cahill et al., 2013), and increase species migration rates in certain areas (Loarie et al., 2009; Malcolm et al., 2002). Climate change can also affect the biology of herpetofauna. For example, global warming affects the sex ratio of hatching turtles (Fuentes et al., 2009). Owing to their biological characteristics, herpetofauna exhibit heightened sensitivity to climate change, possibly contributing to the global decline in their populations (Huey et al., 2012; Seebacher et al., 2015).

**FIGURE 1:**
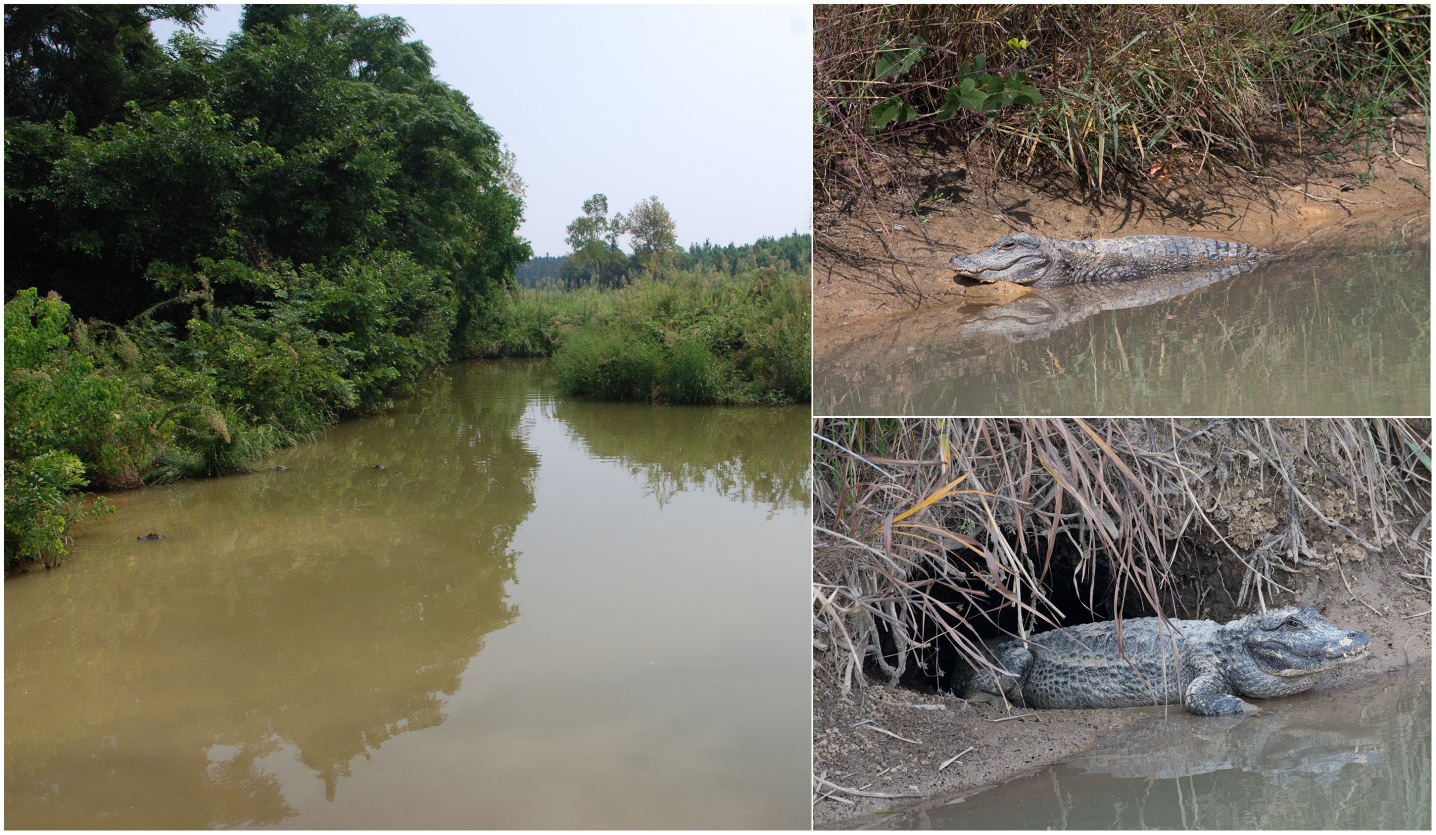
Pictures of the Chinese alligator (Photo taken by Xiaobing Wu in Anhui Chinese Alligator National Nature Reserve).

The critically endangered Chinese alligator (*Alligator sinensis*) (Figure 1) is narrowly distributed in southeastern Anhui Province, China (Pan et al., 2019). As a kind of reptile that is secondary adapted to the aquatic environment, it shows high dependence on climate factors such as temperature and rainfall. The area where Chinese alligators once lived covered almost the entire middle and lower reaches of the Yangtze River (Thorbjarnarson et al., 2002). Habitat suitability decline and fragmentation (Caused by climate change and human influence) are the primary factors contributing to the restricted range of Chinese alligators (Chen et al., 2003; Pan et al., 2019; Thorbjarnarson et al., 2002). While this species originally inhabited various wetlands, continuous habitat loss in recent years has limited its distribution to small, isolated freshwater ponds and canals in the southeast Anhui Province (Jiang & Wu, 2018). Fed mainly on fish, birds, and small mammals, Chinese alligators are at the top of the food chain and play an important role in maintaining the balance of freshwater ecosystems (Livingstone, 2012).

Since species with narrow distributions are more vulnerable to climate change (Schwartz et al., 2006; Thomas, 2010), it is important to understand the implications of climate change on the habitat of range‐restricted Chinese alligators. Furthermore, examining the protective, mitigative measures for Chinese alligators within protected areas is essential.

Because of its simple operation and reliable results, the Maxent model has been widely used in conservation biology research such as biological invasion and endangered species protection. In addition, the Maxent model also shows excellent performance in the simulation of narrow distribution areas of species, such as giant pandas, red pandas, and Crocodile Lizards (Liu et al., 2022; Thapa et al., 2018; Zhang et al., 2022). In this study, we employed a species distribution model (Maxent) to assess changes in habitat suitability for the Chinese alligator in the middle and lower reaches of the Yangtze River amidst climate change. Integrating the minimum investment theory principle, we aimed to understand the distribution pattern of Chinese alligator habitats and identify their priority protection areas under climate change, eventually providing data to support their conservation.

## 2 MATERIALS AND METHODS

### 2.1 Data source and preprocessing

This study, conducted in the middle and lower reaches of the Yangtze River in China (longitude range: 105.00°–122.60°, latitude range: 24.20°–35.30°), employed field monitoring data from June to September in recent years (2018–2023) to assess the suitability of alligator habitats. All the fieldwork was carried out with the permission and accompaniment of Anhui Chinese Alligator National Nature Reserve Administration, and the survey area covered all the current wild habitats of Chinese alligators. Finally, a total of 67 occurrence records (with exact latitude and longitude) of the Chinese alligator were collected by the line transect method.

**FIGURE 2:**
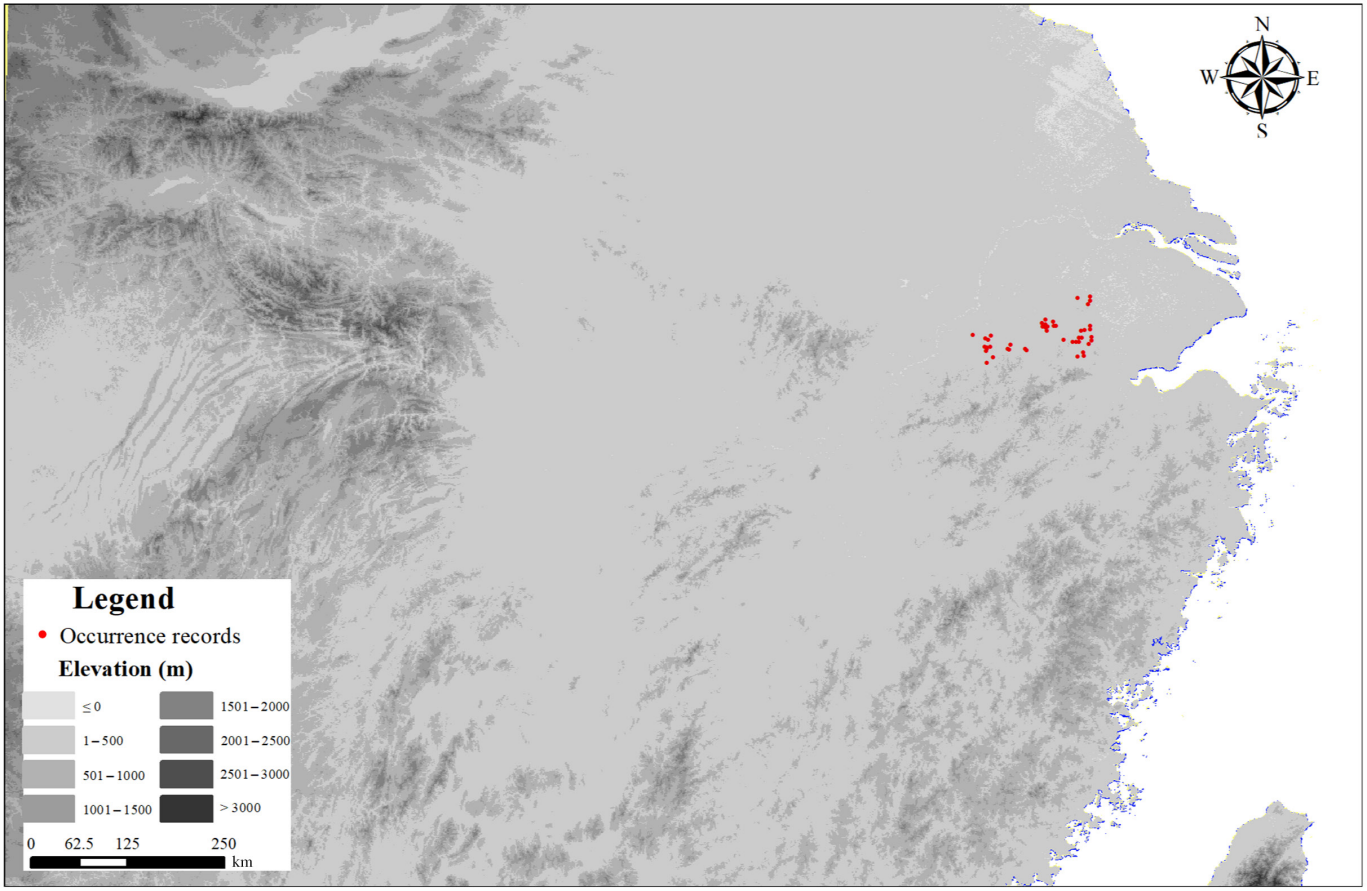
Occurrence records map of the Chinese alligator.

We rigorously screened the environmental and occurrence data to avoid model overfitting. Initially, we used ENMstools to filter the occurrence data, ensuring only one species distribution point per 1 km × 1 km grid (Warren et al., 2010). Then, we retained 46 occurrence records for modeling (Figure 2, Table S1). This study exclusively focused on the impact of climate change on Chinese alligator habitats. We obtained 19 climatic variables from the WorldClim website (http://www.worldclim.org/cmip6_30s), and details of all bioclimatic variables are listed in Table 1. Subsequently, we processed all collected variables in ENMstools, removing environmental variables with high correlation (*r* > .7, Table S2), (Dormann et al., 2013; Yang et al., 2020, 2023). Among the highly correlated variables, we retained factors established as crucial for reptile distribution in a prior study and extreme climatic factors, considering the substantial influence of extreme climates on poikilotherms (Sun et al., 2019). The selected variables included Bio3 (isothermality), Bio5 (maximum temperature of the warmest month), Bio6 (minimum temperature of the coldest month), Bio12 (annual precipitation), Bio15 (precipitation seasonality), and Bio18 (precipitation of the warmest quarter). The selected variables can reflect the annual range and degree of local temperature and the demands for rainfall for Chinese alligators.

**TABLE 1:** Environmental variables used for this study.

Variable groups	Environmental variables	Codes	Data sources	Resolution
Bioclimate	Annual mean temperature	Bio1 (×10°C)	Worldclim 1.4, http://www.worldclim.org/cmip5_30s	~1 km^2^ (30‐s)
	Mean diurnal range of temperature	Bio2 (×10°C)	Worldclim 1.4, http://www.worldclim.org/cmip5_30s	~1 km^2^ (30‐s)
	Isothermality (Bio2/Bio7) (×100)	Bio3	Worldclim 1.4, http://www.worldclim.org/cmip5_30s	~1 km^2^ (30‐s)
	Temperature seasonality (standard deviation × 100)	Bio4	Worldclim 1.4, http://www.worldclim.org/cmip5_30s	~1 km^2^ (30‐s)
	Maximum temperature of warmest month	Bio5 (×10°C)	Worldclim 1.4, http://www.worldclim.org/cmip5_30s	~1 km^2^ (30‐s)
	Minimum temperature of coldest month	Bio6 (×10°C)	Worldclim 1.4, http://www.worldclim.org/cmip5_30s	~1 km^2^ (30‐s)
	Temperature annual range (BIO5–BIO6)	Bio7 (×10°C)	Worldclim 1.4, http://www.worldclim.org/cmip5_30s	~1 km^2^ (30‐s)
	Mean temperature of wettest quarter	Bio8 (×10°C)	Worldclim 1.4, http://www.worldclim.org/cmip5_30s	~1 km^2^ (30‐s)
	Mean temperature of driest quarter	Bio9 (×10°C)	Worldclim 1.4, http://www.worldclim.org/cmip5_30s	~1 km^2^ (30‐s)
	Mean temperature of warmest quarter	Bio10 (×10°C)	Worldclim 1.4, http://www.worldclim.org/cmip5_30s	~1 km^2^ (30‐s)
	Mean temperature of coldest quarter	Bio11 (×10°C)	Worldclim 1.4, http://www.worldclim.org/cmip5_30s	~1 km^2^ (30‐s)
	Annual precipitation	Bio12 (mm)	Worldclim 1.4, http://www.worldclim.org/cmip5_30s	~1 km^2^ (30‐s)
	Precipitation of wettest month	Bio13 (mm)	Worldclim 1.4, http://www.worldclim.org/cmip5_30s	~1 km^2^ (30‐s)
	Precipitation of driest month	Bio14 (mm)	Worldclim 1.4, http://www.worldclim.org/cmip5_30s	~1 km^2^ (30‐s)
	Precipitation seasonality (Coefficient of Variation)	Bio15	Worldclim 1.4, http://www.worldclim.org/cmip5_30s	~1 km^2^ (30‐s)
	Precipitation of wettest quarter	Bio16 (mm)	Worldclim 1.4, http://www.worldclim.org/cmip5_30s	~1 km^2^ (30‐s)
	Precipitation of driest quarter	Bio17 (mm)	Worldclim 1.4, http://www.worldclim.org/cmip5_30s	~1 km^2^ (30‐s)
	Precipitation of warmest quarter	Bio18 (mm)	Worldclim 1.4, http://www.worldclim.org/cmip5_30s	~1 km^2^ (30‐s)
	Precipitation of coldest quarter	Bio19 (mm)	Worldclim 1.4, http://www.worldclim.org/cmip5_30s	~1 km^2^ (30‐s)
Topography	Elevation	ELE (m)	Resource and Environment Science and Data Center, http://www.resdc.cn/DOI/doi.aspx?DOIid=49	~1 km^2^ (30‐s)

*Note*: And the elevation variable is not used for modeling, but only for elevation extraction analysis after modeling.

We used ArcGIS 10.2 for all spatial and statistical analyses. All variables were converted to the ASC format of projected coordinates (Beijing 1954 3 Degree GK CM 108E). To reduce the uncertainties of model prediction, we chose three internationally recognized General Circulation Models (BCC‐CSM2‐MR, IPSL‐CM6A‐LR, and MIROC6) and two Shared Socioeconomic Pathways (SSP126 and SSP585) from the IPCC's Sixth Assessment Report for predictions for the periods 2041–2060 and 2061–2080. The general circulation model is a set of partial differential equations or solutions of fluid mechanics and thermodynamics, which is constructed according to the basic physical laws and used to study and simulate the basic properties of atmospheric circulation or predict its future state changes. Based on the current climate conditions, the climate conditions of different time periods can be obtained by solving these equations so as to achieve the prediction of global climate change. BCC‐CSM2‐MR, IPSL‐CM6A‐LR, and MIROC6 are the three General Circulation Models used more frequently and have better prediction results in the study area (Erskine Ogden & Rejmánek, 2005; Ponce‐Reyes et al., 2017; Wu et al., 2016). SSP126 and SSP585 represent the best‐ and worst‐case scenarios, respectively, reflecting the overall trend of climate change for conservation planning purposes. In order to reduce the model's error in climate predictions differently, we averaged the three GCMs under the two future SSPs in the 2050s and 2070s, and the average layer is used as future climate data for modeling.

### 2.2 Optimization and establishment of the model

To reduce the influence of the calibration region on the calibration result, the method of enclosing rectangles was used to determine the calibration region consistent with the study region (the middle and lower reaches of the Yangtze River, longitude range: 105.00°–122.60°, latitude range: 24.20°–35.30°). The study area covers all potential distribution areas of Chinese alligators. Then we built 1160 candidate models using the kuenm package in R (version: 3.6.3) to prevent overfitting of the model. Firstly, these models were based on a combination of the regularization multiplier (RM, ranging from 0.1 to 6 with an interval of 0.1) and all the feature classes. Next, the distribution data of the Chinese alligators were randomly divided into two groups: 20% were randomly selected as the test set, and the remaining 80% as the training set. The analysis was then performed using MaxEnt.3.4.1 with 10 bootstrap replicates. Assuming that the Chinese alligator population can spread freely in the study area, all species models were developed using the occurrence data and 10,000 random background points representing environmental conditions in the study region (Merow et al., 2013). Other Maxent model parameters were selected as default.

In these candidate models, the parameters with the statistical difference, lowest omission rate, and lowest AICc value were identified as optimal (feature classes: threshold; RM = 1.3) (Cobos et al., 2019). The specific statistical data for model optimization are listed in Table S3. The optimal model was selected as the species distribution model. Finally, the continuous Boyce index (CBI), area under roc curve (AUC), and true skill statistics (TSS) were used as the evaluation index of the model (Allouche et al., 2006; Silva et al., 2017). The habitat suitability threshold for Chinese alligators was calculated according to the maximum specificity and sensitivity (MSS). The MSS method is usually used to calculate the species distribution threshold with only present data (Hijmans et al., 2017).

To understand the overall change in habitat suitability for Chinese alligators, we used the reclass function in ArcGIS 10.2. The MSS threshold indicates a binary threshold for unsuitable and suitable classification of continuous probability maps for species suitability. In addition, *p* > .5 is another reasonable index to distinguish habitat suitability. So we divided their habitats into three categories based on suitability: highly suitable habitat (*p* ≥ .5); poorly suitable (threshold ≤ *p* < .5); unsuitable (0 ≤ *p* < threshold) (Merow et al., 2013; Yang et al., 2023).

### 2.3 Centroid migration and elevation analysis

Centroid migration reflects the overall trends of changes in species distribution patterns. In this study, we divided the research area into small grids of 1 km × 1 km and each grid had a corresponding species distribution probability, which was denoted as *p*. The projection coordinates for each raster were then calculated. Assuming the study area was composed of i rasters, the weight proportion occupied by the *N* ^th^ raster was given by *G*_*n*_ = *P*_*n*_ × *S*_*n*_, where *P*_*n*_ represents the habitat suitability of the species in the *n* ^th^ grid, and *S*_*n*_ represents the area of the *n* ^th^ grid.

The projection coordinates of the center of mass are as follows:

$$X=\frac{\sum_{n=1}^{n}G_{n}X_{n}}{\sum_{n=1}^{n}G_{n}};Y=\frac{\sum_{k=1}^{n}G_{n}Y_{n}}{\sum_{n=1}^{n}G_{n}}.$$

Here, *X*_*n*_ and *Y*_*n*_ represent the longitude and latitude of the *n* ^th^ grid, respectively (He et al., 2010). Additionally, we extracted the habitat elevation of Chinese alligators during various periods, focusing on suitability above the existence threshold to evaluate changes under climate change.

### 2.4 Identification of priority protected areas

Optimizing financial resources has consistently been a practical principle in protected area planning (Naidoo et al., 2006; Watts et al., 2017). Therefore, the area requiring the least costly ideal protection was designated as the priority protection area. Identifying these priority protected areas is crucial in conservation planning for endangered animals. Continuous global climate changes necessitate constant updates to the original planning area to ensure alignment with evolving habitat conditions.

In this study, we employed the Marxan model to identify priority protected areas of Chinese alligators amid global climate change. The decline in habitat suitability is a primary factor in the recent decline of the wild Chinese alligator population (Pan et al., 2019; Thorbjarnarson et al., 2002), emphasizing the need to focus conservation efforts on ensuring a suitable habitat. Initially, we divided the study area into 1 km × 1 km grids, considering each grid's area as the planned unit cost. We then considered the habitat suitability of the Chinese alligator (Output by the Maxent model) corresponding to each net as the account value for that unit, and the unsuitable region (*p* < threshold) is assigned a value of 0. Subsequently, the Marxan model was applied to identify priority protected areas, and the results for each climate scenario were superimposed. The displayed value of the planning unit is the number of climate scenarios in which the planning unit is identified as a priority protection area.

Additionally, the protective effect of protected areas was evaluated to protect the Anhui Chinese Alligator National Nature Reserve by fitting the extent of protected areas and the Marxan results.

## 3 RESULTS

### 3.1 Modeling and habitat change analysis

The continuous Boyd index, AUC, and TSS indicated that the simulation results of all models reached an excellent level, and all values are presented in Table S4. The model validation showed that the predicted results were excellent and suitable for further analyses.

**FIGURE 3:**
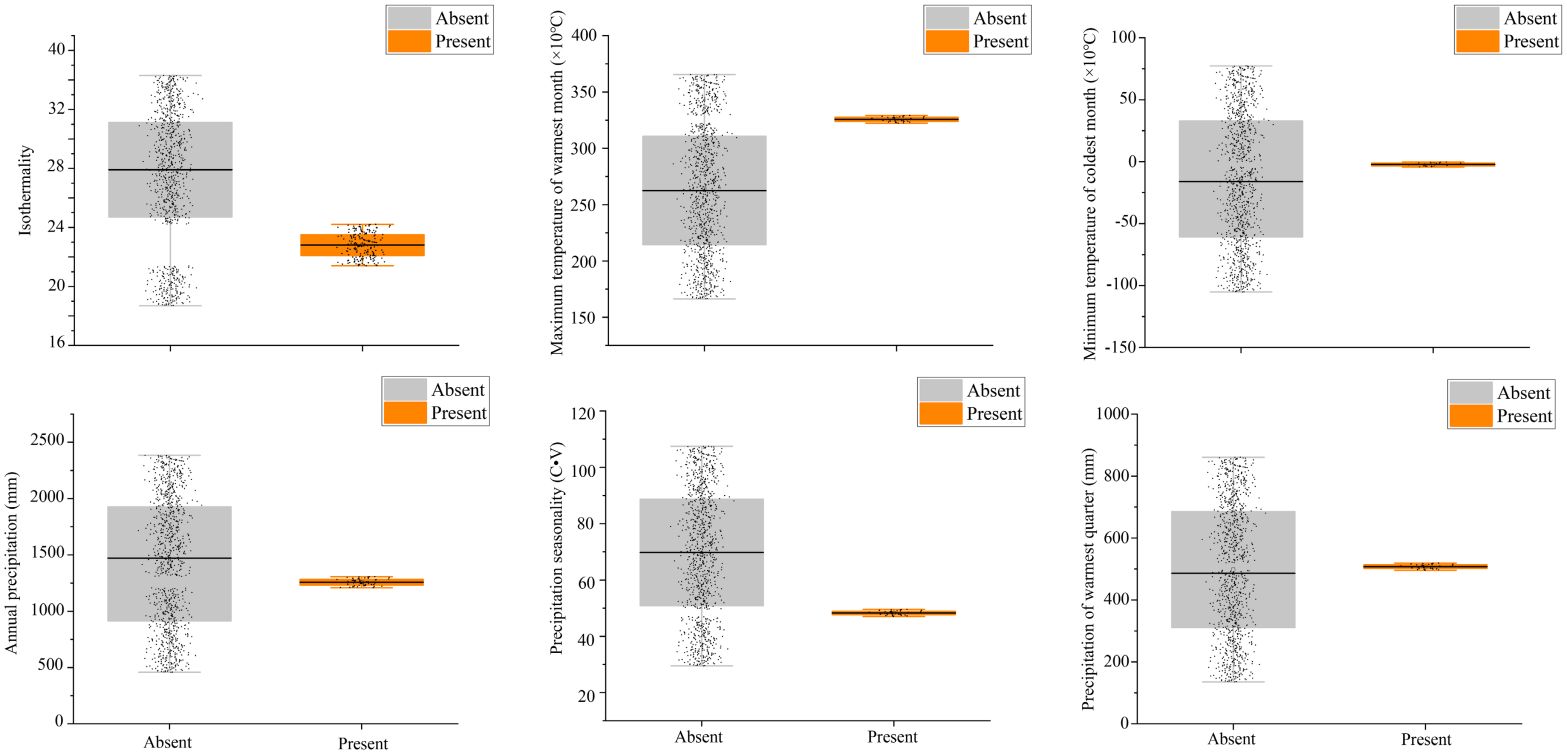
Responses of Chinese alligators to various climatic factors. Suitable and unsuitable indicate the variable range of suitable habitat (highly suitable habitat and poorly suitable habitat) and unsuitable habitat of Chinese alligators, respectively.

The jackknife analysis results showed that the variables with high importance to the model were Bio6 (minimum temperature of coldest month) and Bio15 (rainfall seasonality), with a total importance of 69.4%. Specifically, Bio6 and Bio15 had importance rates of 49.5% and 19.9%, respectively (see Table 2 for the importance of individual predictors). The overall and optimal range of each variable in the study area (suitability threshold) are presented in Table S5 and Figure 3. For the variables with the highest contributions (Bio6 and Bio15), the suitable distribution range of Chinese alligators was very narrow.

**TABLE 2:** Analysis of bioclimatic variable importance of *Alligator sinensis*.

Variables	Percent importance (%)
Bio6	49.5
Bio15	19.9
Bio18	9.7
Bio5	8.2
Bio12	7.2
Bio3	5.5

The MSS method yielded a threshold value of 0.34, and subsequent calculations determined the areas for four habitat types. The outcomes revealed a relatively small habitat area for Chinese alligators. Specifically, unsuitable habitats covered 1,863,140.77 km^2^, constituting 99.0021% of the study areas. The total proportion of the other two habitat types was 0.9979%, with a highly suitable habitat of 0.8011% (See Table 2 for details). This shows that their habitats are relatively suitable despite the narrow current distribution of Chinese alligators.

**FIGURE 4:**
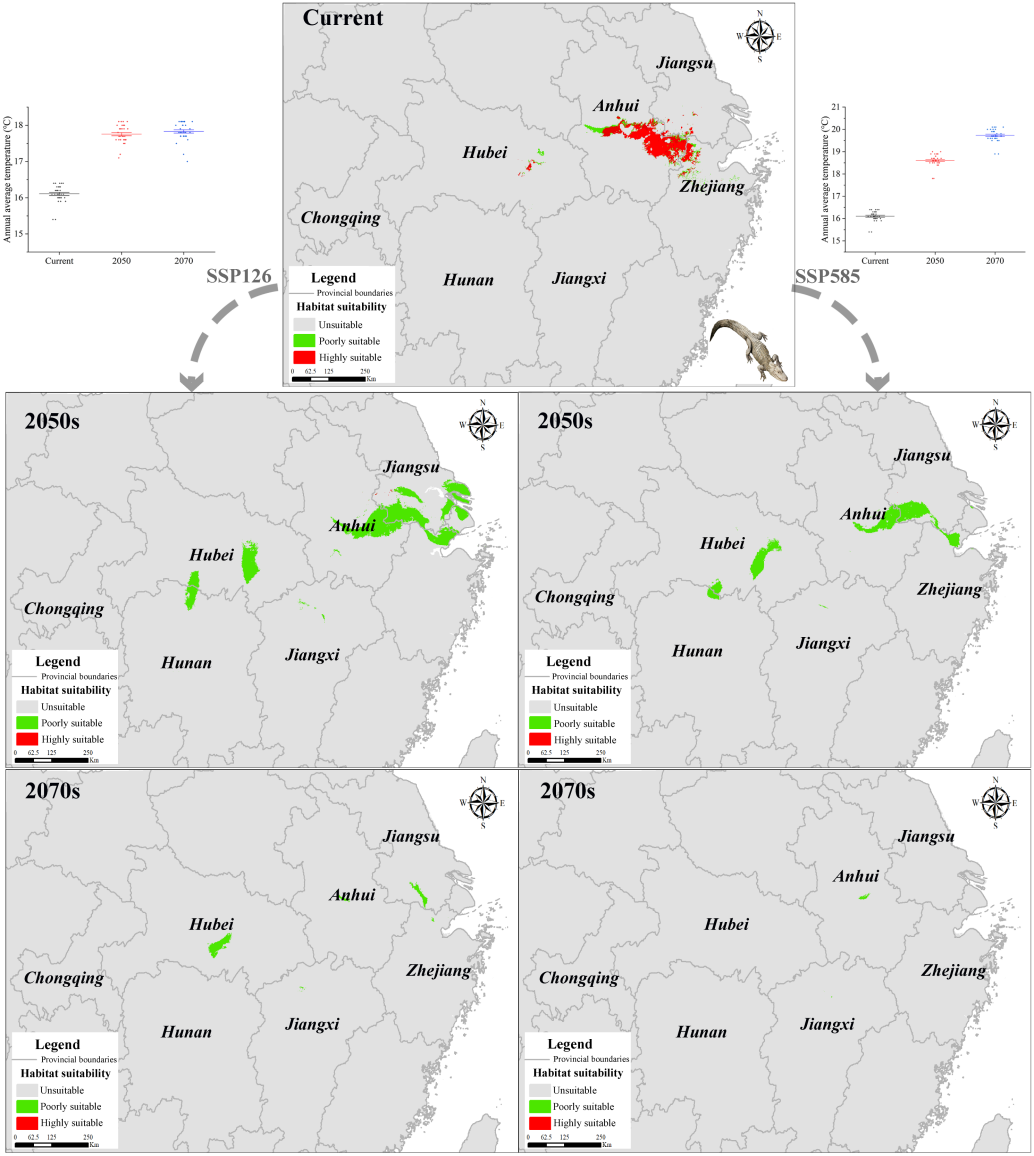
Current and future habitat simulations of the Chinese alligator.

The results showed that the most suitable habitats for Chinese alligators were primarily located in southeast Anhui, as depicted in Figure 4. Their future range simulation results projected a decrease in suitable habitat area under all scenarios by 2050s and 2070s, especially in suitable habitats (Figure 4, Table 3). Table 3 outlines the anticipated degree of change in the four habitat areas of the Chinese alligator.

**TABLE 3:** Future habitat composition of *Alligator sinensis* in China.

Year	SSPs	Area km^2^			Percentage (%)		
		Unsuitable	Poorly suitable	Suitable	Unsuitable	Poorly suitable	Suitable
Current	–	1,863,140.77	3704.88	15,075.59	99.0021	0.1969	0.8011
2041–2060	SSP126	1,854,804.57	25,735.32	49.58	98.6299	1.3675	0.0027
	SSP585	1,865,278.15	18,141.13	2.73	99.0359	0.9640	0.0001
2061–2080	SSP126	1,878,256.86	3664.38	0	99.8053	0.1947	0
	SSP585	1,881,623.52	297.72	0	99.9842	0.0158	0

Forecasts indicate a reduction in the suitable habitat of the Chinese alligator to varying degrees, with a near‐total loss of suitable habitat. Moreover, the future distribution area of Chinese alligators tends toward fragmentation, intensifying over time. This trend is partly associated with the SSPs, with SSP585 showing the most severe impact. Under SSP585, the area of suitable habitat experiences the greatest decrease, particularly in highly suitable habitats, where the proportion approaches zero by 2040–2060. In general, the habitat of Chinese alligators under ssp585 decreased by 17.93%–40.69% more than that under ssp126.

### 3.2 Centroid migration and elevation analysis

The barycenter migration results showed that the distribution center of Chinese alligators will move westward in the future (Figure S1). The extent of barycenter migration is expected to range from 51.5535 to 55.5565 km by the 2050s. This westward shift would persist, and by the 2070s, the distribution center is anticipated to migrate from 149.9303 to 165.7141 km (Tables S6 and S7). Elevation analysis showed that the habitat elevation of Chinese alligators would not change considerably in the future, and the average altitude of the most suitable distribution for Chinese alligators would be <100 m (Figure S2).

### 3.3 Identification of priority conservation areas

**FIGURE 5:**
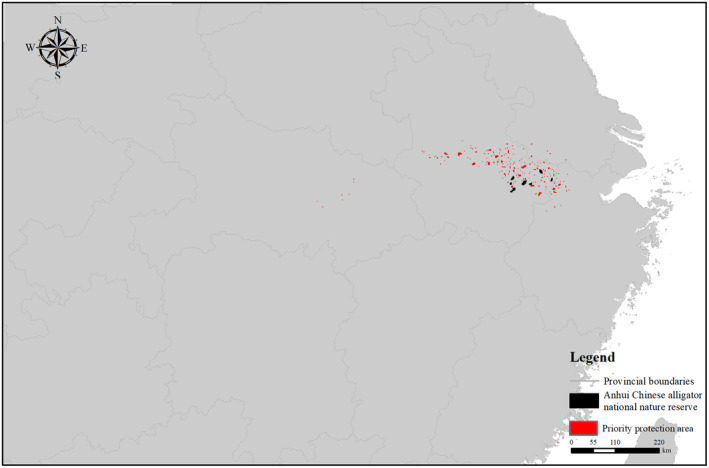
Identification of current priority conservation areas.

The Marxan analysis showed that 8732 units covering 8706.17 km^2^ are priority conservation areas in the current scenario (Figure 5). Notably, 21.73% of the Anhui Chinese Alligator National Nature Reserve fell within these priority protection areas. In the four future scenarios, 17.88 km^2^, representing 8.82% of the protected area, were designated as priority protection areas.

Optimal solutions from Marxan were superimposed across the five scenarios (current and future). An area could be identified as a priority protection area in up to three scenarios, with a total of 4 units covering an area of 4 km^2^ (Figure S3). Additional statistical values can be found in Table S8.

## 4 DISCUSSION

Global climate is changing at an unprecedented rate (Adedeji, 2014; Turner et al., 2018). These changes are mainly manifested through global warming, shifts in rainfall patterns, and the increased frequency of climate extremes (IPCC, 2023). In China, the climate change trend mirrors that of the world, with a slightly higher warming rate. Projections for China indicate a continued rise in surface temperature and precipitation over the next 20–100 years (Guo et al., 2017; Wen et al., 2016). The effects of these changes on biodiversity are often devastating, particularly in tropical and narrow regions (Laurance et al., 2009; Wilson et al., 2006). According to IPCC and Worldclim data (https://worldclim.org) (IPCC, 2023), the climate of the current distribution area of Chinese alligators will change considerably in the future (Figure S4). These changes have led to a loss of suitable habitats for Chinese alligators.

Habitat loss is the main reason for the decline of the wild Chinese alligator population (Zhou et al., 2012). Historically, Chinese alligators have been distributed throughout China. However, climate change and other factors have led to the continuous loss of its habitat (Pan et al., 2019), resulting in its narrow and fragmented distribution pattern. The jackknife results showed that Bio6 and Bio15 are the two most important variables affecting the distribution of Chinese alligators. Bio6 reflects extremely low local temperatures, particularly during the coldest month when Chinese alligators enter a state of hibernation. Temperature during hibernation is critical for their survival; excessively low temperatures can lead to frostbite, while high temperatures can shorten the hibernation cycle (Gao et al., 2015; Turbill & Prior, 2016). However, adequate hibernation time is crucial for the alligator's growth and development (Lin et al., 2020). Meanwhile, Chinese alligators, adapted to secondary aquatic life, have distinct demands related to their aquatic environment. Notably, their water requirements exhibit a strong seasonality. During the breeding season (warmer months), increased rainfall is essential to meet the elevated humidity needs for successful egg incubation (López‐Luna et al., 2015; Murray et al., 2020). However, hibernating alligators are slow and unable to cope with rising water levels caused by excessive winter rainfall, which may eventually cause the alligators to suffocate and die (Chen et al., 2003). Balancing these seasonal water requirements is crucial for the species' overall well‐being.

Contrary to a common trend in many species responding to climate change, such as migration toward higher latitudes and elevations (Jiang et al., 2020; Pauchard et al., 2016), Chinese alligators have not exhibited this phenomenon previously. The decrease in habitat suitability in the east resulted in the westward migration of the center of mass points within the distribution area of Chinese alligators. The unique habitat requirements of Chinese alligators resulted in their characteristic distribution at lower altitudes. The MaxEnt analysis results showed that the habitat suitability of the Chinese alligators is a gradual reduction and fragmentation process under climate change (Figure 4, Table 3). This pattern is consistent with climate change responses observed in other species, such as *Ailuropoda melanoleuca*, *Bufo gargarizans*, and *Sclerophrys perreti* (Gong et al., 2017; Nneji et al., 2020; Yang et al., 2023).

To protect the wild Chinese alligator populations effectively, the Chinese government established the Anhui Chinese Alligator National Nature Reserve with on‐site protection of the Chinese alligator. Our research showed that this reserve plays an important role in conserving Chinese alligators and their response to climate change. However, protection gaps may exist, especially when 76.24% of the reserve area is not designated as a priority protected area. With climate change, this proportion will continue to increase. Therefore, identifying the priority protected areas of Chinese alligators under climate change is particularly crucial.

Priority conservation areas identified by systematic conservation planning tools, such as Marxan, can protect species' habitats at a minimal cost (Liang et al., 2018; Watts et al., 2017), and these areas provide refuge for the species (Mi et al., 2023). Our results can guide the establishment of nature reserves, contributing to the effective conservation of Chinese alligators by protecting their suitable habitats more comprehensively and efficiently.

In response to climate change threats, species typically adapt through two primary mechanisms: seeking suitable habitats and adjusting through their inherent plasticity (Mainwaring et al., 2017; Merilä & Hendry, 2014). To properly assess the threat of climate change to Chinese alligators, future research should delve into individual plasticity under climate change (Richter et al., 2012; Seebacher et al., 2015). This study will be able to comprehensively assess the population risk of Chinese alligators under climate change by incorporating plasticity data (the research we are doing) and finally provide suggestions for the protection of Chinese alligators.

## AUTHOR CONTRIBUTIONS

**Liuyang Yang:** Conceptualization (lead); data curation (equal); formal analysis (lead); investigation (equal); software (equal); visualization (equal); writing – original draft (lead). **Jiangnan Ling:** Data curation (equal); formal analysis (supporting); investigation (equal). **Lilei Lu:** Formal analysis (supporting); investigation (equal). **Dongsheng Zang:** Formal analysis (supporting); investigation (equal). **Yunzhen Zhu:** Formal analysis (supporting); investigation (equal). **Song Zhang:** Data curation (equal); resources (equal). **Yongkang Zhou:** Data curation (equal); resources (equal). **Pingsi Yi:** Formal analysis (equal); resources (equal). **En Li:** Methodology (equal); supervision (equal). **Tao Pan:** Methodology (equal); project administration (equal); supervision (equal); writing – review and editing (equal). **Xiaobing Wu:** Funding acquisition (equal); investigation (equal); methodology (equal); project administration (equal); supervision (equal); writing – review and editing (equal).

## CONFLICT OF INTEREST STATEMENT

The authors have no competing interests to declare.

## Supporting information


Appendix S1.

## Data Availability

The occurrence records of Chinese alligators are listed in the attachment. The environment variables were derived from official websites (refer to Table 1 for the specific download sites).
