# Supplementary material for: Identification of suitable habitats and priority conservation areas under climate change scenarios for the Chinese alligator (Alligator sinensis)
Source: Ecol Evol. 2024 May 30;14(6):e11477. doi: 10.1002/ece3.11477 (PMC11137492; doi:10.1002/ece3.11477)
Supplement: Supplementary file 1 — Appendix S1. [file ECE3-14-e11477-s001.docx]

**Table S1 Data on the distribution of *Alligator* s*inensis* used in this study**

| **species** | **longitude** | **latitude** |
| --- | --- | --- |
| *Alligator sinensis* | 1496409.460 | 3455161.329 |
| *Alligator sinensis* | 1499103.631 | 3469505.943 |
| *Alligator sinensis* | 1505324.239 | 3446609.692 |
| *Alligator sinensis* | 1502677.957 | 3474546.245 |
| *Alligator sinensis* | 1524541.762 | 3457839.174 |
| *Alligator sinensis* | 1528113.725 | 3462890.040 |
| *Alligator sinensis* | 1549131.359 | 3455533.222 |
| *Alligator sinensis* | 1574937.583 | 3481668.208 |
| *Alligator sinensis* | 1573507.913 | 3495651.561 |
| *Alligator sinensis* | 1586508.314 | 3487559.837 |
| *Alligator sinensis* | 1596473.747 | 3469748.601 |
| *Alligator sinensis* | 1609034.746 | 3466338.780 |
| *Alligator sinensis* | 1615020.094 | 3448099.024 |
| *Alligator sinensis* | 1613064.420 | 3466761.311 |
| *Alligator sinensis* | 1617084.708 | 3467184.419 |
| *Alligator sinensis* | 1616592.180 | 3471852.874 |
| *Alligator sinensis* | 1620620.561 | 3472278.916 |
| *Alligator sinensis* | 1623085.339 | 3448945.249 |
| *Alligator sinensis* | 1619631.217 | 3481604.989 |
| *Alligator sinensis* | 1622593.333 | 3453614.415 |
| *Alligator sinensis* | 1615148.167 | 3523584.308 |
| *Alligator sinensis* | 1623656.175 | 3482033.275 |
| *Alligator sinensis* | 1629673.727 | 3463796.834 |
| *Alligator sinensis* | 1633197.653 | 3468895.839 |
| *Alligator sinensis* | 1632697.724 | 3473565.596 |
| *Alligator sinensis* | 1631697.273 | 3482893.613 |
| *Alligator sinensis* | 1631195.550 | 3487563.072 |
| *Alligator sinensis* | 1628175.087 | 3515555.328 |
| *Alligator sinensis* | 1631681.271 | 3520648.553 |
| *Alligator sinensis* | 1631172.899 | 3525317.529 |
| *Alligator sinensis* | 1479246.567 | 3475880.483 |
| *Alligator sinensis* | 1493969.076 | 3460332.551 |
| *Alligator sinensis* | 1497099.729 | 3439674.617 |
| *Alligator sinensis* | 1495512.366 | 3471004.314 |
| *Alligator sinensis* | 1496635.847 | 3459380.261 |
| *Alligator sinensis* | 1497060.289 | 3457348.339 |
| *Alligator sinensis* | 1501921.797 | 3460576.352 |
| *Alligator sinensis* | 1526276.592 | 3456417.000 |
| *Alligator sinensis* | 1547240.768 | 3457498.828 |
| *Alligator sinensis* | 1569201.460 | 3486359.859 |
| *Alligator sinensis* | 1569173.137 | 3491036.970 |
| *Alligator sinensis* | 1572989.221 | 3485683.249 |
| *Alligator sinensis* | 1573477.908 | 3489191.910 |
| *Alligator sinensis* | 1575711.355 | 3486961.728 |
| *Alligator sinensis* | 1582988.839 | 3493446.226 |
| *Alligator sinensis* | 1584217.128 | 3488053.774 |

**Table S2 Correlation between variables**

| Variables | bio3 | bio5 | bio6 | bio12 | bio15 | bio18 |
| --- | --- | --- | --- | --- | --- | --- |
| bio3 | 0 | -0.067891244 | -0.083671856 | 0.00819037 | 0.378080606 | 0.092179651 |
| bio5 | 0 | 0 | 0.565059358 | -0.012718125 | -0.167473541 | -0.356880342 |
| bio6 | 0 | 0 | 0 | 0.515010708 | -0.493743392 | 0.093592172 |
| bio12 | 0 | 0 | 0 | 0 | -0.586772821 | 0.69554302 |
| bio15 | 0 | 0 | 0 | 0 | 0 | -0.144446648 |
| bio18 | 0 | 0 | 0 | 0 | 0 | 0 |

**Table S3 Performance statistics for all candidate models**

| Models | | Pval  pROC | Omission rate at 5% | AICc | Delta  AICc | num parameters |
| --- | --- | --- | --- | --- | --- | --- |
| RM | FC |  |  |  |  |  |
| 0.1 | l | 0 | 0 | 1126.471932 | 118.7951992 | 6 |
| 0.1 | q | 0 | 0.111111111 | 1104.339519 | 96.66278664 | 6 |
| 0.1 | p | 0 | 0 | 1085.356884 | 77.68015168 | 11 |
| 0.1 | t | 0 | 0.111111111 | NA | NA | 82 |
| 0.1 | h | 0 | 0 | NA | NA | 68 |
| 0.1 | lq | 0 | 0.111111111 | 1015.133125 | 7.456392795 | 12 |
| 0.1 | lp | 0 | 0 | 1096.10131 | 88.42457705 | 17 |
| 0.1 | lt | 0 | 0.111111111 | NA | NA | 82 |
| 0.1 | lh | 0 | 0 | NA | NA | 71 |
| 0.1 | qp | 0 | 0.111111111 | 1021.355365 | 13.6786329 | 13 |
| 0.1 | qt | 0 | 0.111111111 | NA | NA | 86 |
| 0.1 | qh | 0 | 0 | NA | NA | 63 |
| 0.1 | pt | 0 | 0.111111111 | NA | NA | 82 |
| 0.1 | ph | 0 | 0 | NA | NA | 56 |
| 0.1 | th | 0 | 0.111111111 | NA | NA | 86 |
| 0.1 | lqp | 0 | 0.111111111 | 1040.029328 | 32.35259501 | 18 |
| 0.1 | lqt | 0 | 0.111111111 | NA | NA | 86 |
| 0.1 | lqh | 0 | 0 | NA | NA | 62 |
| 0.1 | lpt | 0 | 0.111111111 | NA | NA | 82 |
| 0.1 | lph | 0 | 0 | NA | NA | 57 |
| 0.1 | qpt | 0 | 0.111111111 | NA | NA | 86 |
| 0.1 | qph | 0 | 0 | NA | NA | 59 |
| 0.1 | qth | 0 | 0.111111111 | NA | NA | 86 |
| 0.1 | pth | 0 | 0.111111111 | NA | NA | 86 |
| 0.1 | lqpt | 0 | 0.111111111 | NA | NA | 86 |
| 0.1 | lqph | 0 | 0 | NA | NA | 59 |
| 0.1 | lqth | 0 | 0.111111111 | NA | NA | 86 |
| 0.1 | lpth | 0 | 0.111111111 | NA | NA | 86 |
| 0.1 | lqpth | 0 | 0.111111111 | NA | NA | 86 |
| 0.2 | l | 0 | 0 | 1126.445513 | 118.7687804 | 6 |
| 0.2 | q | 0 | 0.111111111 | 1105.073115 | 97.39638263 | 6 |
| 0.2 | p | 0 | 0 | 1085.886261 | 78.20952829 | 10 |
| 0.2 | t | 0 | 0.111111111 | NA | NA | 57 |
| 0.2 | h | 0 | 0 | NA | NA | 57 |
| 0.2 | lq | 0 | 0.111111111 | 1032.915639 | 25.23890681 | 11 |
| 0.2 | lp | 0 | 0 | 1091.780953 | 84.10422088 | 14 |
| 0.2 | lt | 0 | 0.111111111 | NA | NA | 57 |
| 0.2 | lh | 0 | 0 | NA | NA | 53 |
| 0.2 | qp | 0 | 0.111111111 | 1035.913458 | 28.23672552 | 10 |
| 0.2 | qt | 0 | 0.111111111 | NA | NA | 62 |
| 0.2 | qh | 0 | 0 | NA | NA | 60 |
| 0.2 | pt | 0 | 0.111111111 | NA | NA | 60 |
| 0.2 | ph | 0 | 0 | 1318.40605 | 310.7293173 | 36 |
| 0.2 | th | 0 | 0 | NA | NA | 65 |
| 0.2 | lqp | 0 | 0.111111111 | 1045.603797 | 37.92706489 | 13 |
| 0.2 | lqt | 0 | 0.111111111 | NA | NA | 62 |
| 0.2 | lqh | 0 | 0 | NA | NA | 53 |
| 0.2 | lpt | 0 | 0.111111111 | NA | NA | 60 |
| 0.2 | lph | 0 | 0 | 1377.289705 | 369.612972 | 37 |
| 0.2 | qpt | 0 | 0.111111111 | NA | NA | 62 |
| 0.2 | qph | 0 | 0 | 2239.759465 | 1232.082733 | 42 |
| 0.2 | qth | 0 | 0 | NA | NA | 66 |
| 0.2 | pth | 0 | 0.111111111 | NA | NA | 67 |
| 0.2 | lqpt | 0 | 0.111111111 | NA | NA | 62 |
| 0.2 | lqph | 0 | 0 | 1376.213017 | 368.5362841 | 37 |
| 0.2 | lqth | 0 | 0 | NA | NA | 66 |
| 0.2 | lpth | 0 | 0 | NA | NA | 65 |
| 0.2 | lqpth | 0 | 0 | NA | NA | 66 |
| 0.3 | l | 0 | 0 | 1126.462203 | 118.7854702 | 6 |
| 0.3 | q | 0 | 0.111111111 | 1106.279636 | 98.60290387 | 6 |
| 0.3 | p | 0 | 0 | 1104.167193 | 96.4904605 | 12 |
| 0.3 | t | 0 | 0.111111111 | 1655.601369 | 647.9246362 | 40 |
| 0.3 | h | 0 | 0 | NA | NA | 56 |
| 0.3 | lq | 0 | 0.111111111 | 1043.836877 | 36.16014415 | 8 |
| 0.3 | lp | 0 | 0 | 1107.910123 | 100.23339 | 13 |
| 0.3 | lt | 0 | 0.111111111 | 4963.350828 | 3955.674096 | 44 |
| 0.3 | lh | 0 | 0 | 2938.119572 | 1930.44284 | 43 |
| 0.3 | qp | 0 | 0.111111111 | 1042.859005 | 35.18227202 | 8 |
| 0.3 | qt | 0 | 0.111111111 | 2894.782727 | 1887.105995 | 43 |
| 0.3 | qh | 0 | 0 | 1903.16507 | 895.488337 | 41 |
| 0.3 | pt | 0 | 0.111111111 | 2202.373831 | 1194.697098 | 42 |
| 0.3 | ph | 0 | 0 | 1145.672538 | 137.995806 | 30 |
| 0.3 | th | 0 | 0.111111111 | NA | NA | 47 |
| 0.3 | lqp | 0 | 0.111111111 | 1042.430382 | 34.75364982 | 8 |
| 0.3 | lqt | 0 | 0.111111111 | 2892.964057 | 1885.287325 | 43 |
| 0.3 | lqh | 0 | 0 | 1902.789893 | 895.1131602 | 41 |
| 0.3 | lpt | 0 | 0.111111111 | 4962.654896 | 3954.978164 | 44 |
| 0.3 | lph | 0 | 0 | 1143.342704 | 135.6659713 | 30 |
| 0.3 | qpt | 0 | 0.111111111 | NA | NA | 46 |
| 0.3 | qph | 0 | 0 | 1282.15317 | 274.4764372 | 35 |
| 0.3 | qth | 0 | 0.111111111 | NA | NA | 46 |
| 0.3 | pth | 0 | 0.111111111 | NA | NA | 47 |
| 0.3 | lqpt | 0 | 0.111111111 | NA | NA | 46 |
| 0.3 | lqph | 0 | 0 | 1144.131955 | 136.4552226 | 30 |
| 0.3 | lqth | 0 | 0.111111111 | NA | NA | 46 |
| 0.3 | lpth | 0 | 0.111111111 | NA | NA | 49 |
| 0.3 | lqpth | 0 | 0.111111111 | NA | NA | 45 |
| 0.4 | l | 0 | 0 | 1126.522756 | 118.8460231 | 6 |
| 0.4 | q | 0 | 0.111111111 | 1107.726523 | 100.0497902 | 6 |
| 0.4 | p | 0 | 0 | 1109.897367 | 102.2206341 | 10 |
| 0.4 | t | 0 | 0.111111111 | 1136.140972 | 128.4642398 | 31 |
| 0.4 | h | 0 | 0 | NA | NA | 48 |
| 0.4 | lq | 0 | 0.111111111 | 1050.665394 | 42.9886615 | 7 |
| 0.4 | lp | 0 | 0 | 1119.830287 | 112.153555 | 13 |
| 0.4 | lt | 0 | 0.111111111 | 1354.824399 | 347.147667 | 37 |
| 0.4 | lh | 0 | 0 | 1071.189677 | 63.51294418 | 24 |
| 0.4 | qp | 0 | 0.111111111 | 1050.832899 | 43.15616639 | 7 |
| 0.4 | qt | 0 | 0.111111111 | 1183.611701 | 175.9349683 | 33 |
| 0.4 | qh | 0 | 0 | 1081.94862 | 74.27188763 | 25 |
| 0.4 | pt | 0 | 0.111111111 | 1212.329002 | 204.6522691 | 34 |
| 0.4 | ph | 0 | 0 | 1093.787298 | 86.11056534 | 26 |
| 0.4 | th | 0 | 0 | 4977.447296 | 3969.770563 | 44 |
| 0.4 | lqp | 0 | 0.111111111 | 1050.566751 | 42.89001847 | 7 |
| 0.4 | lqt | 0 | 0.111111111 | 1527.172057 | 519.4953245 | 39 |
| 0.4 | lqh | 0 | 0 | 1104.206936 | 96.5302035 | 27 |
| 0.4 | lpt | 0 | 0.111111111 | 1353.205122 | 345.5283898 | 37 |
| 0.4 | lph | 0 | 0 | 1081.880487 | 74.20375411 | 25 |
| 0.4 | qpt | 0 | 0.111111111 | 1296.44631 | 288.7695778 | 36 |
| 0.4 | qph | 0 | 0 | 1082.334837 | 74.65810496 | 25 |
| 0.4 | qth | 0 | 0.111111111 | 1296.78156 | 289.1048271 | 36 |
| 0.4 | pth | 0 | 0.111111111 | 1526.051393 | 518.3746609 | 39 |
| 0.4 | lqpt | 0 | 0.111111111 | 1296.44631 | 288.7695778 | 36 |
| 0.4 | lqph | 0 | 0 | 1081.567096 | 73.89036328 | 25 |
| 0.4 | lqth | 0 | 0.111111111 | 1427.739935 | 420.0632025 | 38 |
| 0.4 | lpth | 0 | 0.111111111 | 1353.213012 | 345.5362795 | 37 |
| 0.4 | lqpth | 0 | 0.111111111 | 1526.071008 | 518.3942753 | 39 |
| 0.5 | l | 0 | 0 | 1126.624546 | 118.9478135 | 6 |
| 0.5 | q | 0 | 0.111111111 | 1109.375922 | 101.6991892 | 6 |
| 0.5 | p | 0 | 0 | 1125.287428 | 117.6106955 | 12 |
| 0.5 | t | 0 | 0.111111111 | 1045.153727 | 37.47699411 | 24 |
| 0.5 | h | 0 | 0 | 2262.995418 | 1255.318685 | 42 |
| 0.5 | lq | 0 | 0.111111111 | 1055.188979 | 47.51224659 | 7 |
| 0.5 | lp | 0 | 0 | 1114.904491 | 107.2277589 | 9 |
| 0.5 | lt | 0 | 0.111111111 | 1104.224069 | 96.54733671 | 29 |
| 0.5 | lh | 0 | 0 | 1061.3899 | 53.71316721 | 22 |
| 0.5 | qp | 0 | 0.111111111 | 1055.282311 | 47.60557854 | 7 |
| 0.5 | qt | 0 | 0.111111111 | 1076.425099 | 68.74836636 | 27 |
| 0.5 | qh | 0 | 0 | 1039.058508 | 31.3817751 | 19 |
| 0.5 | pt | 0 | 0.111111111 | 1088.134143 | 80.45741 | 28 |
| 0.5 | ph | 0 | 0 | 1071.305439 | 63.62870667 | 23 |
| 0.5 | th | 0 | 0 | 1090.513485 | 82.83675248 | 28 |
| 0.5 | lqp | 0 | 0.111111111 | 1055.084564 | 47.40783149 | 7 |
| 0.5 | lqt | 0 | 0.111111111 | 1120.690594 | 113.0138619 | 30 |
| 0.5 | lqh | 0 | 0 | 1078.586835 | 70.9101023 | 24 |
| 0.5 | lpt | 0 | 0.111111111 | 1103.290315 | 95.61358243 | 29 |
| 0.5 | lph | 0 | 0 | 1079.539238 | 71.86250584 | 24 |
| 0.5 | qpt | 0 | 0.111111111 | 1103.61018 | 95.93344734 | 29 |
| 0.5 | qph | 0 | 0 | 1070.067762 | 62.39102943 | 23 |
| 0.5 | qth | 0 | 0.111111111 | 1089.541584 | 81.86485184 | 28 |
| 0.5 | pth | 0 | 0 | 1140.504888 | 132.8281557 | 31 |
| 0.5 | lqpt | 0 | 0.111111111 | 1103.61018 | 95.93344734 | 29 |
| 0.5 | lqph | 0 | 0 | 1078.544235 | 70.86750265 | 24 |
| 0.5 | lqth | 0 | 0.111111111 | 1120.99671 | 113.3199774 | 30 |
| 0.5 | lpth | 0 | 0.111111111 | 1162.987388 | 155.3106557 | 32 |
| 0.5 | lqpth | 0 | 0 | 1163.190824 | 155.5140916 | 32 |
| 0.6 | l | 0 | 0 | 1126.75972 | 119.0829876 | 6 |
| 0.6 | q | 0 | 0.111111111 | 1111.224078 | 103.5473451 | 6 |
| 0.6 | p | 0 | 0 | 1118.657821 | 110.9810885 | 9 |
| 0.6 | t | 0 | 0.111111111 | 1017.892753 | 10.21602028 | 20 |
| 0.6 | h | 0 | 0 | 1480.015342 | 472.3386091 | 38 |
| 0.6 | lq | 0 | 0.111111111 | 1059.422472 | 51.74573924 | 7 |
| 0.6 | lp | 0 | 0 | 1115.109009 | 107.4322767 | 8 |
| 0.6 | lt | 0 | 0.111111111 | 1057.785808 | 50.1090759 | 25 |
| 0.6 | lh | 0 | 0 | 1035.205028 | 27.52829517 | 17 |
| 0.6 | qp | 0 | 0.111111111 | 1059.533425 | 51.85669249 | 7 |
| 0.6 | qt | 0 | 0.111111111 | 1049.167977 | 41.49124489 | 24 |
| 0.6 | qh | 0 | 0 | 1032.612463 | 24.93573031 | 17 |
| 0.6 | pt | 0 | 0.111111111 | 1080.832952 | 73.1562199 | 27 |
| 0.6 | ph | 0 | 0 | 1077.016895 | 69.34016207 | 23 |
| 0.6 | th | 0 | 0 | 1059.261459 | 51.5847263 | 25 |
| 0.6 | lqp | 0 | 0.111111111 | 1059.272388 | 51.59565518 | 7 |
| 0.6 | lqt | 0 | 0.111111111 | 1069.024031 | 61.34729856 | 26 |
| 0.6 | lqh | 0 | 0 | 1052.158555 | 44.48182228 | 20 |
| 0.6 | lpt | 0 | 0.111111111 | 1080.815525 | 73.1387923 | 27 |
| 0.6 | lph | 0 | 0 | 1069.300041 | 61.62330877 | 22 |
| 0.6 | qpt | 0 | 0.111111111 | 1080.677144 | 73.00041193 | 27 |
| 0.6 | qph | 0 | 0 | 1058.63486 | 50.95812736 | 21 |
| 0.6 | qth | 0 | 0.111111111 | 1058.978533 | 51.30180008 | 25 |
| 0.6 | pth | 0 | 0.111111111 | 1069.05992 | 61.38318767 | 26 |
| 0.6 | lqpt | 0 | 0.111111111 | 1080.677144 | 73.00041193 | 27 |
| 0.6 | lqph | 0 | 0 | 1058.678046 | 51.00131397 | 21 |
| 0.6 | lqth | 0 | 0.111111111 | 1058.113279 | 50.43654644 | 25 |
| 0.6 | lpth | 0 | 0.111111111 | 1094.29665 | 86.61991709 | 28 |
| 0.6 | lqpth | 0 | 0.111111111 | 1080.9662 | 73.28946779 | 27 |
| 0.7 | l | 0 | 0 | 1126.940123 | 119.2633906 | 6 |
| 0.7 | q | 0 | 0.111111111 | 1113.191133 | 105.5144009 | 6 |
| 0.7 | p | 0 | 0 | 1113.819776 | 106.143044 | 7 |
| 0.7 | t | 0 | 0.111111111 | 1021.273859 | 13.59712636 | 20 |
| 0.7 | h | 0 | 0 | 1928.885332 | 921.2085999 | 41 |
| 0.7 | lq | 0 | 0 | 1066.570851 | 58.89411865 | 8 |
| 0.7 | lp | 0 | 0 | 1113.866663 | 106.1899302 | 7 |
| 0.7 | lt | 0 | 0.111111111 | 1073.85164 | 66.17490757 | 26 |
| 0.7 | lh | 0 | 0 | 1053.396502 | 45.71976941 | 19 |
| 0.7 | qp | 0 | 0 | 1063.531595 | 55.85486205 | 7 |
| 0.7 | qt | 0 | 0.111111111 | 1021.710007 | 14.0332746 | 20 |
| 0.7 | qh | 0 | 0 | 1042.060483 | 34.38375027 | 18 |
| 0.7 | pt | 0 | 0.111111111 | 1063.209052 | 55.53231901 | 25 |
| 0.7 | ph | 0 | 0 | 1067.293343 | 59.61661096 | 21 |
| 0.7 | th | 0 | 0 | 1086.04597 | 78.36923734 | 27 |
| 0.7 | lqp | 0 | 0 | 1063.326629 | 55.64989679 | 7 |
| 0.7 | lqt | 0 | 0.111111111 | 1074.020986 | 66.3442533 | 26 |
| 0.7 | lqh | 0 | 0 | 1056.997169 | 49.3204368 | 20 |
| 0.7 | lpt | 0 | 0.111111111 | 1073.979953 | 66.3032205 | 26 |
| 0.7 | lph | 0 | 0 | 1036.562582 | 28.88584916 | 16 |
| 0.7 | qpt | 0 | 0.111111111 | 1074.415324 | 66.73859102 | 26 |
| 0.7 | qph | 0 | 0 | 1050.077799 | 42.40106685 | 19 |
| 0.7 | qth | 0 | 0 | 1053.435247 | 45.75851469 | 24 |
| 0.7 | pth | 0 | 0.111111111 | 1073.972233 | 66.29550017 | 26 |
| 0.7 | lqpt | 0 | 0.111111111 | 1074.415324 | 66.73859102 | 26 |
| 0.7 | lqph | 0 | 0 | 1056.523027 | 48.84629464 | 20 |
| 0.7 | lqth | 0 | 0.111111111 | 1035.425104 | 27.74837189 | 22 |
| 0.7 | lpth | 0 | 0.111111111 | 1073.127507 | 65.4507745 | 26 |
| 0.7 | lqpth | 0 | 0.111111111 | 1073.565534 | 65.88880114 | 26 |
| 0.8 | l | 0 | 0 | 1127.146228 | 119.4694958 | 6 |
| 0.8 | q | 0 | 0.111111111 | 1115.231694 | 107.5549616 | 6 |
| 0.8 | p | 0 | 0 | 1111.778334 | 104.1016013 | 6 |
| 0.8 | t | 0 | 0.111111111 | 1018.480196 | 10.80346392 | 19 |
| 0.8 | h | 0 | 0 | 1416.441738 | 408.7650052 | 37 |
| 0.8 | lq | 0 | 0 | 1070.579827 | 62.90309441 | 8 |
| 0.8 | lp | 0 | 0 | 1117.784087 | 110.1073541 | 7 |
| 0.8 | lt | 0 | 0.111111111 | 1040.287067 | 32.61033491 | 22 |
| 0.8 | lh | 0 | 0 | 1039.913262 | 32.23652939 | 16 |
| 0.8 | qp | 0 | 0 | 1067.381478 | 59.70474534 | 7 |
| 0.8 | qt | 0 | 0.111111111 | 1018.539151 | 10.86241834 | 19 |
| 0.8 | qh | 0 | 0 | 1036.560111 | 28.883378 | 16 |
| 0.8 | pt | 0 | 0.111111111 | 1048.615729 | 40.93899616 | 23 |
| 0.8 | ph | 0 | 0 | 1072.152209 | 64.47547625 | 21 |
| 0.8 | th | 0 | 0 | 1066.436951 | 58.76021866 | 25 |
| 0.8 | lqp | 0 | 0 | 1067.217945 | 59.54121255 | 7 |
| 0.8 | lqt | 0 | 0.111111111 | 1057.133288 | 49.45655571 | 24 |
| 0.8 | lqh | 0 | 0 | 1042.48438 | 34.80764776 | 17 |
| 0.8 | lpt | 0 | 0.111111111 | 1067.484551 | 59.80781894 | 25 |
| 0.8 | lph | 0 | 0 | 1045.381576 | 37.70484313 | 17 |
| 0.8 | qpt | 0 | 0.111111111 | 1078.726978 | 71.0502456 | 26 |
| 0.8 | qph | 0 | 0 | 1048.646735 | 40.97000234 | 18 |
| 0.8 | qth | 0 | 0 | 1024.378433 | 16.70170059 | 20 |
| 0.8 | pth | 0 | 0.111111111 | 1066.680486 | 59.00375323 | 25 |
| 0.8 | lqpt | 0 | 0.111111111 | 1078.609752 | 70.93301958 | 26 |
| 0.8 | lqph | 0 | 0 | 1048.622607 | 40.94587413 | 18 |
| 0.8 | lqth | 0 | 0.111111111 | 1055.969224 | 48.29249195 | 24 |
| 0.8 | lpth | 0 | 0.111111111 | 1055.802119 | 48.12538651 | 24 |
| 0.8 | lqpth | 0 | 0.111111111 | 1066.178102 | 58.50136952 | 25 |
| 0.9 | l | 0 | 0 | 1127.37619 | 119.6994578 | 6 |
| 0.9 | q | 0 | 0.111111111 | 1117.306214 | 109.6294819 | 6 |
| 0.9 | p | 0 | 0 | 1109.699922 | 102.0231894 | 5 |
| 0.9 | t | 0 | 0 | 1022.256905 | 14.58017208 | 19 |
| 0.9 | h | 0 | 0 | 1222.271824 | 214.5950919 | 32 |
| 0.9 | lq | 0 | 0 | 1074.164437 | 66.48770401 | 8 |
| 0.9 | lp | 0 | 0 | 1120.957652 | 113.2809192 | 7 |
| 0.9 | lt | 0 | 0.111111111 | 1043.567111 | 35.89037819 | 22 |
| 0.9 | lh | 0 | 0 | 1043.170976 | 35.49424399 | 16 |
| 0.9 | qp | 0 | 0 | 1070.98816 | 63.31142766 | 7 |
| 0.9 | qt | 0 | 0.111111111 | 1021.844332 | 14.1675991 | 19 |
| 0.9 | qh | 0 | 0 | 1027.873867 | 20.19713406 | 13 |
| 0.9 | pt | 0 | 0.111111111 | 1052.310906 | 44.63417396 | 23 |
| 0.9 | ph | 0 | 0 | 1094.399097 | 86.72236455 | 23 |
| 0.9 | th | 0 | 0 | 1050.889817 | 43.21308491 | 23 |
| 0.9 | lqp | 0 | 0 | 1070.84908 | 63.17234766 | 7 |
| 0.9 | lqt | 0 | 0.111111111 | 1060.706907 | 53.03017421 | 24 |
| 0.9 | lqh | 0 | 0 | 1041.424328 | 33.74759581 | 16 |
| 0.9 | lpt | 0 | 0.111111111 | 1061.236302 | 53.55956904 | 24 |
| 0.9 | lph | 0 | 0 | 1049.479443 | 41.80271055 | 17 |
| 0.9 | qpt | 0 | 0.111111111 | 1061.482878 | 53.80614586 | 24 |
| 0.9 | qph | 0 | 0 | 1053.371713 | 45.69498091 | 18 |
| 0.9 | qth | 0 | 0 | 1027.10678 | 19.43004761 | 20 |
| 0.9 | pth | 0 | 0.111111111 | 1051.066072 | 43.3893394 | 23 |
| 0.9 | lqpt | 0 | 0.111111111 | 1061.482878 | 53.80614586 | 24 |
| 0.9 | lqph | 0 | 0 | 1052.71336 | 45.03662799 | 18 |
| 0.9 | lqth | 0 | 0.111111111 | 1034.25445 | 26.57771799 | 21 |
| 0.9 | lpth | 0 | 0.111111111 | 1069.645976 | 61.96924328 | 25 |
| 0.9 | lqpth | 0 | 0.111111111 | 1059.500795 | 51.82406279 | 24 |
| 1 | l | 0 | 0 | 1127.652177 | 119.9754448 | 6 |
| 1.0 | q | 0 | 0.111111111 | 1119.404509 | 111.7277764 | 6 |
| 1.0 | p | 0 | 0 | 1115.14995 | 107.4732174 | 6 |
| 1.0 | t | 0 | 0 | 1019.571459 | 11.89472688 | 18 |
| 1.0 | h | 0 | 0 | 1500.117385 | 492.4406521 | 38 |
| 1.0 | lq | 0 | 0 | 1074.74684 | 67.07010757 | 7 |
| 1.0 | lp | 0 | 0 | 1120.165281 | 112.4885486 | 6 |
| 1.0 | lt | 0 | 0.111111111 | 1039.328919 | 31.65218617 | 21 |
| 1.0 | lh | 0 | 0 | 1033.580808 | 25.90407594 | 13 |
| 1.0 | qp | 0 | 0 | 1074.474794 | 66.79806151 | 7 |
| 1.0 | qt | 0 | 0.111111111 | 1025.266616 | 17.58988377 | 19 |
| 1.0 | qh | 0 | 0 | 1036.545402 | 28.86866943 | 14 |
| 1.0 | pt | 0 | 0.111111111 | 1032.657495 | 24.98076269 | 20 |
| 1.0 | ph | 0 | 0 | 1072.777922 | 65.10118977 | 20 |
| 1.0 | th | 0 | 0 | 1054.355036 | 46.67830303 | 23 |
| 1.0 | lqp | 0 | 0 | 1074.333255 | 66.65652279 | 7 |
| 1.0 | lqt | 0 | 0.111111111 | 1063.950314 | 56.27358159 | 24 |
| 1.0 | lqh | 0 | 0 | 1056.083037 | 48.40630457 | 18 |
| 1.0 | lpt | 0 | 0.111111111 | 1039.867262 | 32.19052901 | 21 |
| 1.0 | lph | 0 | 0 | 1054.497095 | 46.82036295 | 17 |
| 1.0 | qpt | 0 | 0.111111111 | 1039.944768 | 32.26803588 | 21 |
| 1.0 | qph | 0 | 0 | 1038.125031 | 30.44829878 | 14 |
| 1.0 | qth | 0 | 0 | 1036.730601 | 29.05386805 | 21 |
| 1.0 | pth | 0 | 0.111111111 | 1045.697144 | 38.02041145 | 22 |
| 1.0 | lqpt | 0 | 0.111111111 | 1033.172669 | 25.49593664 | 20 |
| 1.0 | lqph | 0 | 0 | 1052.413207 | 44.73647488 | 17 |
| 1.0 | lqth | 0 | 0.111111111 | 1030.074379 | 22.39764627 | 20 |
| 1.0 | lpth | 0 | 0.111111111 | 1072.541472 | 64.86473905 | 25 |
| 1.0 | lqpth | 0 | 0.111111111 | 1072.446207 | 64.76947448 | 25 |
| 1.1 | l | 0 | 0 | 1127.949903 | 120.2731701 | 6 |
| 1.1 | q | 0 | 0.111111111 | 1121.49755 | 113.820818 | 6 |
| 1.1 | p | 0 | 0 | 1116.311191 | 108.6344584 | 6 |
| 1.1 | t | 0 | 0 | 1017.002689 | 9.325956166 | 17 |
| 1.1 | h | 0 | 0 | 1258.686054 | 251.0093211 | 33 |
| 1.1 | lq | 0 | 0 | 1078.045973 | 70.36924076 | 7 |
| 1.1 | lp | 0 | 0 | 1120.348743 | 112.6720102 | 6 |
| 1.1 | lt | 0 | 0.111111111 | 1022.778844 | 15.10211135 | 18 |
| 1.1 | lh | 0 | 0 | 1037.5717 | 29.89496798 | 13 |
| 1.1 | qp | 0 | 0 | 1077.809272 | 70.13253961 | 7 |
| 1.1 | qt | 0 | 0.111111111 | 1022.534527 | 14.85779481 | 18 |
| 1.1 | qh | 0 | 0 | 1032.394787 | 24.71805445 | 12 |
| 1.1 | pt | 0 | 0.111111111 | 1029.143924 | 21.46719161 | 19 |
| 1.1 | ph | 0 | 0 | 1056.5458 | 48.86906773 | 16 |
| 1.1 | th | 0 | 0 | 1041.201871 | 33.52513888 | 21 |
| 1.1 | lqp | 0 | 0 | 1077.63447 | 69.95773738 | 7 |
| 1.1 | lqt | 0 | 0.111111111 | 1057.642044 | 49.96531197 | 23 |
| 1.1 | lqh | 0 | 0 | 1053.994154 | 46.3174213 | 17 |
| 1.1 | lpt | 0 | 0.111111111 | 1042.78506 | 35.10832748 | 21 |
| 1.1 | lph | 0 | 0 | 1043.106296 | 35.42956341 | 14 |
| 1.1 | qpt | 0 | 0.111111111 | 1029.638155 | 21.96142236 | 19 |
| 1.1 | qph | 0 | 0 | 1041.58805 | 33.91131718 | 14 |
| 1.1 | qth | 0 | 0 | 1032.796213 | 25.11948091 | 20 |
| 1.1 | pth | 0 | 0.111111111 | 1056.825953 | 49.14922072 | 23 |
| 1.1 | lqpt | 0 | 0.111111111 | 1029.570251 | 21.89351806 | 19 |
| 1.1 | lqph | 0 | 0 | 1046.117412 | 38.44067941 | 15 |
| 1.1 | lqth | 0 | 0 | 1026.479195 | 18.80246252 | 19 |
| 1.1 | lpth | 0 | 0.111111111 | 1075.361969 | 67.68523633 | 25 |
| 1.1 | lqpth | 0 | 0.111111111 | 1041.244473 | 33.56774069 | 21 |
| 1.2 | l | 0 | 0 | 1128.264598 | 120.5878658 | 6 |
| 1.2 | q | 0 | 0 | 1123.566631 | 115.889898 | 6 |
| 1.2 | p | 0 | 0 | 1122.019479 | 114.3427469 | 7 |
| 1.2 | t | 0 | 0 | 1014.390469 | 6.713736837 | 16 |
| 1.2 | h | 0 | 0 | 1332.664072 | 324.9873397 | 35 |
| 1.2 | lq | 0 | 0 | 1081.16862 | 73.49188724 | 7 |
| 1.2 | lp | 0 | 0 | 1120.618637 | 112.9419044 | 6 |
| 1.2 | lt | 0 | 0.111111111 | 1025.430555 | 17.7538223 | 18 |
| 1.2 | lh | 0 | 0 | 1033.317901 | 25.6411688 | 11 |
| 1.2 | qp | 0 | 0 | 1083.987992 | 76.31125908 | 8 |
| 1.2 | qt | 0 | 0.111111111 | 1025.226261 | 17.54952809 | 18 |
| 1.2 | qh | 0 | 0 | 1040.678782 | 33.00204941 | 13 |
| 1.2 | pt | 0 | 0.111111111 | 1025.845254 | 18.16852147 | 18 |
| 1.2 | ph | 0 | 0 | 1105.919695 | 98.24296241 | 23 |
| 1.2 | th | 0 | 0 | 1043.711261 | 36.03452892 | 21 |
| 1.2 | lqp | 0 | 0 | 1083.891855 | 76.21512288 | 8 |
| 1.2 | lqt | 0 | 0.111111111 | 1037.792502 | 30.11576965 | 20 |
| 1.2 | lqh | 0 | 0 | 1047.994012 | 40.31727989 | 15 |
| 1.2 | lpt | 0 | 0.111111111 | 1032.366997 | 24.69026426 | 19 |
| 1.2 | lph | 0 | 0 | 1046.277324 | 38.60059173 | 14 |
| 1.2 | qpt | 0 | 0.111111111 | 1032.331079 | 24.65434654 | 19 |
| 1.2 | qph | 0 | 0 | 1041.296147 | 33.61941497 | 13 |
| 1.2 | qth | 0 | 0 | 1023.387435 | 15.71070222 | 18 |
| 1.2 | pth | 0 | 0.111111111 | 1044.065622 | 36.38888902 | 21 |
| 1.2 | lqpt | 0 | 0.111111111 | 1038.7911 | 31.11436794 | 20 |
| 1.2 | lqph | 0 | 0 | 1049.673149 | 41.99641641 | 15 |
| 1.2 | lqth | 0 | 0 | 1023.382049 | 15.70531653 | 18 |
| 1.2 | lpth | 0 | 0.111111111 | 1068.467063 | 60.79033002 | 24 |
| 1.2 | lqpth | 0 | 0.111111111 | 1037.135721 | 29.4589886 | 20 |
| 1.3 | l | 0 | 0 | 1128.616572 | 120.939839 | 6 |
| 1.3 | q | 0 | 0 | 1125.614783 | 117.9380505 | 6 |
| 1.3 | p | 0 | 0 | 1124.206443 | 116.5297106 | 7 |
| 1.3 | t | 0 | 0 | 1007.676733 | 0 | 14 |
| 1.3 | h | 0 | 0 | 1299.828374 | 292.151642 | 34 |
| 1.3 | lq | 0 | 0 | 1084.171729 | 76.49499681 | 7 |
| 1.3 | lp | 0 | 0 | 1120.844314 | 113.1675813 | 6 |
| 1.3 | lt | 0 | 0.111111111 | 1028.245066 | 20.56833333 | 18 |
| 1.3 | lh | 0 | 0 | 1048.698173 | 41.02144057 | 14 |
| 1.3 | qp | 0 | 0 | 1087.80775 | 80.13101738 | 8 |
| 1.3 | qt | 0 | 0.111111111 | 1022.588138 | 14.91140563 | 17 |
| 1.3 | qh | 0 | 0 | 1044.618844 | 36.94211148 | 13 |
| 1.3 | pt | 0 | 0.222222222 | 1023.20043 | 15.52369754 | 17 |
| 1.3 | ph | 0 | 0 | 1087.246253 | 79.56952001 | 20 |
| 1.3 | th | 0 | 0 | 1032.982599 | 25.30586631 | 19 |
| 1.3 | lqp | 0 | 0 | 1087.586798 | 79.91006506 | 8 |
| 1.3 | lqt | 0 | 0.111111111 | 1047.672804 | 39.99607114 | 21 |
| 1.3 | lqh | 0 | 0 | 1051.523878 | 43.84714596 | 15 |
| 1.3 | lpt | 0 | 0.222222222 | 1035.228635 | 27.55190234 | 19 |
| 1.3 | lph | 0 | 0 | 1050.036475 | 42.35974274 | 14 |
| 1.3 | qpt | 0 | 0.222222222 | 1029.268978 | 21.59224535 | 18 |
| 1.3 | qph | 0 | 0 | 1048.770581 | 41.09384827 | 14 |
| 1.3 | qth | 0 | 0.111111111 | 1020.516761 | 12.84002815 | 17 |
| 1.3 | pth | 0 | 0.111111111 | 1054.574539 | 46.89780631 | 22 |
| 1.3 | lqpt | 0 | 0.222222222 | 1041.598493 | 33.92176055 | 20 |
| 1.3 | lqph | 0 | 0 | 1053.483998 | 45.8072655 | 15 |
| 1.3 | lqth | 0 | 0 | 1020.790706 | 13.11397398 | 17 |
| 1.3 | lpth | 0 | 0.111111111 | 1046.699751 | 39.02301883 | 21 |
| 1.3 | lqpth | 0 | 0.111111111 | 1046.828193 | 39.15146098 | 21 |
| 1.4 | l | 0 | 0 | 1128.994225 | 121.3174922 | 6 |
| 1.4 | q | 0 | 0 | 1127.620842 | 119.9441091 | 6 |
| 1.4 | p | 0 | 0 | 1122.882408 | 115.2056756 | 6 |
| 1.4 | t | 0 | 0 | 1010.180945 | 2.504212641 | 14 |
| 1.4 | h | 0 | 0 | 1342.23883 | 334.5620979 | 35 |
| 1.4 | lq | 0 | 0 | 1087.042582 | 79.36584965 | 7 |
| 1.4 | lp | 0 | 0 | 1121.141684 | 113.4649515 | 6 |
| 1.4 | lt | 0 | 0.111111111 | 1031.21706 | 23.540327 | 18 |
| 1.4 | lh | 0 | 0 | 1051.908353 | 44.23162047 | 14 |
| 1.4 | qp | 0 | 0 | 1091.355409 | 83.67867647 | 8 |
| 1.4 | qt | 0 | 0.111111111 | 1020.309618 | 12.63288586 | 16 |
| 1.4 | qh | 0 | 0 | 1048.695518 | 41.01878527 | 13 |
| 1.4 | pt | 0 | 0.222222222 | 1020.947374 | 13.2706411 | 16 |
| 1.4 | ph | 0 | 0 | 1091.745556 | 84.06882369 | 20 |
| 1.4 | th | 0 | 0 | 1024.135545 | 16.45881202 | 17 |
| 1.4 | lqp | 0 | 0 | 1091.076415 | 83.39968251 | 8 |
| 1.4 | lqt | 0 | 0.111111111 | 1043.715731 | 36.03899801 | 20 |
| 1.4 | lqh | 0 | 0 | 1038.640774 | 30.96404169 | 11 |
| 1.4 | lpt | 0 | 0.222222222 | 1026.954593 | 19.27786 | 17 |
| 1.4 | lph | 0 | 0 | 1053.847495 | 46.17076271 | 14 |
| 1.4 | qpt | 0 | 0.222222222 | 1026.729009 | 19.05227624 | 17 |
| 1.4 | qph | 0 | 0 | 1057.129047 | 49.45231419 | 15 |
| 1.4 | qth | 0 | 0.111111111 | 1017.966246 | 10.28951354 | 16 |
| 1.4 | pth | 0 | 0.111111111 | 1043.06357 | 35.38683755 | 20 |
| 1.4 | lqpt | 0 | 0.222222222 | 1032.357346 | 24.68061351 | 18 |
| 1.4 | lqph | 0 | 0 | 1062.056865 | 54.38013277 | 16 |
| 1.4 | lqth | 0 | 0 | 1028.888029 | 21.2112966 | 18 |
| 1.4 | lpth | 0 | 0.111111111 | 1042.815802 | 35.13906916 | 20 |
| 1.4 | lqpth | 0 | 0.111111111 | 1036.603953 | 28.92722068 | 19 |
| 1.5 | l | 0 | 0 | 1129.393431 | 121.7166988 | 6 |
| 1.5 | q | 0 | 0 | 1129.600815 | 121.924082 | 6 |
| 1.5 | p | 0 | 0 | 1123.718488 | 116.0417551 | 6 |
| 1.5 | t | 0 | 0 | 1012.779425 | 5.102692652 | 14 |
| 1.5 | h | 0 | 0 | 1208.978003 | 201.3012701 | 30 |
| 1.5 | lq | 0 | 0 | 1089.772188 | 82.09545573 | 7 |
| 1.5 | lp | 0 | 0 | 1121.45146 | 113.7747273 | 6 |
| 1.5 | lt | 0 | 0.111111111 | 1028.77975 | 21.10301733 | 17 |
| 1.5 | lh | 0 | 0 | 1051.286663 | 43.6099304 | 13 |
| 1.5 | qp | 0 | 0 | 1091.747603 | 84.07087098 | 7 |
| 1.5 | qt | 0 | 0.111111111 | 1023.362316 | 15.6855833 | 16 |
| 1.5 | qh | 0 | 0 | 1049.156572 | 41.47983901 | 12 |
| 1.5 | pt | 0 | 0.222222222 | 1023.750883 | 16.07415 | 16 |
| 1.5 | ph | 0 | 0 | 1082.448794 | 74.77206198 | 18 |
| 1.5 | th | 0 | 0 | 1016.490868 | 8.814135971 | 15 |
| 1.5 | lqp | 0 | 0 | 1091.376326 | 83.69959372 | 7 |
| 1.5 | lqt | 0 | 0.111111111 | 1034.494929 | 26.81819679 | 18 |
| 1.5 | lqh | 0 | 0 | 1054.209175 | 46.532442 | 14 |
| 1.5 | lpt | 0 | 0.222222222 | 1029.826736 | 22.15000337 | 17 |
| 1.5 | lph | 0 | 0 | 1045.50588 | 37.8291472 | 11 |
| 1.5 | qpt | 0 | 0.111111111 | 1029.773631 | 22.0968981 | 17 |
| 1.5 | qph | 0 | 0 | 1056.470009 | 48.793276 | 14 |
| 1.5 | qth | 0 | 0.111111111 | 1020.589061 | 12.91232859 | 16 |
| 1.5 | pth | 0 | 0.111111111 | 1027.895823 | 20.21909095 | 17 |
| 1.5 | lqpt | 0 | 0.111111111 | 1035.383069 | 27.70633623 | 18 |
| 1.5 | lqph | 0 | 0 | 1052.142517 | 44.46578402 | 13 |
| 1.5 | lqth | 0 | 0 | 1026.051165 | 18.3744322 | 17 |
| 1.5 | lpth | 0 | 0.111111111 | 1039.535734 | 31.8590017 | 19 |
| 1.5 | lqpth | 0 | 0.111111111 | 1033.544431 | 25.86769852 | 18 |
| 1.6 | l | 0 | 0 | 1129.810486 | 122.1337532 | 6 |
| 1.6 | q | 0 | 0 | 1131.556635 | 123.8799022 | 6 |
| 1.6 | p | 0 | 0 | 1124.602168 | 116.9254358 | 6 |
| 1.6 | t | 0 | 0 | 1015.481145 | 7.804412209 | 14 |
| 1.6 | h | 0 | 0 | 1213.614433 | 205.9377006 | 30 |
| 1.6 | lq | 0 | 0 | 1092.386168 | 84.70943531 | 7 |
| 1.6 | lp | 0 | 0 | 1121.742481 | 114.0657483 | 6 |
| 1.6 | lt | 0 | 0.111111111 | 1021.945206 | 14.26847317 | 15 |
| 1.6 | lh | 0 | 0 | 1054.809686 | 47.13295343 | 13 |
| 1.6 | qp | 0 | 0 | 1094.234515 | 86.55778217 | 7 |
| 1.6 | qt | 0 | 0.111111111 | 1017.26753 | 9.590797588 | 14 |
| 1.6 | qh | 0 | 0 | 1057.234605 | 49.55787247 | 13 |
| 1.6 | pt | 0 | 0.222222222 | 1026.632457 | 18.95572428 | 16 |
| 1.6 | ph | 0 | 0 | 1086.781416 | 79.10468308 | 18 |
| 1.6 | th | 0 | 0 | 1018.704562 | 11.02782925 | 15 |
| 1.6 | lqp | 0 | 0 | 1093.970065 | 86.29333231 | 7 |
| 1.6 | lqt | 0 | 0.111111111 | 1026.890017 | 19.21328454 | 16 |
| 1.6 | lqh | 0 | 0 | 1057.523413 | 49.84668052 | 14 |
| 1.6 | lpt | 0 | 0.222222222 | 1032.9602 | 25.28346758 | 17 |
| 1.6 | lph | 0 | 0 | 1056.936804 | 49.26007197 | 13 |
| 1.6 | qpt | 0 | 0.111111111 | 1032.863037 | 25.18630497 | 17 |
| 1.6 | qph | 0 | 0 | 1051.949793 | 44.27306099 | 12 |
| 1.6 | qth | 0 | 0.111111111 | 1018.574635 | 10.89790231 | 15 |
| 1.6 | pth | 0 | 0.111111111 | 1030.39103 | 22.71429771 | 17 |
| 1.6 | lqpt | 0 | 0.111111111 | 1038.454115 | 30.77738289 | 18 |
| 1.6 | lqph | 0 | 0 | 1051.881828 | 44.20509562 | 12 |
| 1.6 | lqth | 0 | 0 | 1028.772203 | 21.09547049 | 17 |
| 1.6 | lpth | 0 | 0.111111111 | 1036.092551 | 28.4158184 | 18 |
| 1.6 | lqpth | 0 | 0.111111111 | 1042.126172 | 34.4494391 | 19 |
| 1.7 | l | 0 | 0 | 1130.261864 | 122.5851319 | 6 |
| 1.7 | q | 0 | 0 | 1133.453732 | 125.776999 | 6 |
| 1.7 | p | 0 | 0 | 1125.46695 | 117.790217 | 6 |
| 1.7 | t | 0 | 0 | 1018.272847 | 10.5961143 | 14 |
| 1.7 | h | 0 | 0 | 1287.156913 | 279.4801808 | 33 |
| 1.7 | lq | 0 | 0 | 1094.862288 | 87.18555509 | 7 |
| 1.7 | lp | 0 | 0 | 1122.115713 | 114.4389802 | 6 |
| 1.7 | lt | 0 | 0.111111111 | 1024.977896 | 17.30116352 | 15 |
| 1.7 | lh | 0 | 0 | 1062.660178 | 54.98344585 | 14 |
| 1.7 | qp | 0 | 0 | 1096.758843 | 89.08211024 | 7 |
| 1.7 | qt | 0 | 0.111111111 | 1020.318571 | 12.64183871 | 14 |
| 1.7 | qh | 0 | 0 | 1069.666804 | 61.99007157 | 15 |
| 1.7 | pt | 0 | 0.222222222 | 1024.303646 | 16.62691356 | 15 |
| 1.7 | ph | 0 | 0 | 1090.948037 | 83.27130423 | 18 |
| 1.7 | th | 0 | 0 | 1025.70467 | 18.0279377 | 16 |
| 1.7 | lqp | 0 | 0 | 1096.391929 | 88.71519641 | 7 |
| 1.7 | lqt | 0 | 0.111111111 | 1029.954582 | 22.27784956 | 16 |
| 1.7 | lqh | 0 | 0 | 1061.548595 | 53.87186292 | 14 |
| 1.7 | lpt | 0 | 0.111111111 | 1030.436262 | 22.75952952 | 16 |
| 1.7 | lph | 0 | 0 | 1053.056988 | 45.38025539 | 11 |
| 1.7 | qpt | 0 | 0.111111111 | 1030.364016 | 22.68728391 | 16 |
| 1.7 | qph | 0 | 0 | 1051.880546 | 44.20381368 | 11 |
| 1.7 | qth | 0 | 0.111111111 | 1021.157916 | 13.48118388 | 15 |
| 1.7 | pth | 0 | 0.111111111 | 1033.117313 | 25.44058039 | 17 |
| 1.7 | lqpt | 0 | 0.111111111 | 1035.492046 | 27.81531347 | 17 |
| 1.7 | lqph | 0 | 0 | 1055.501027 | 47.82429461 | 12 |
| 1.7 | lqth | 0 | 0 | 1031.562387 | 23.8856544 | 17 |
| 1.7 | lpth | 0 | 0.111111111 | 1044.875144 | 37.19841139 | 19 |
| 1.7 | lqpth | 0 | 0.111111111 | 1044.759766 | 37.08303307 | 19 |
| 1.8 | l | 0 | 0 | 1130.716466 | 123.0397333 | 6 |
| 1.8 | q | 0 | 0 | 1135.323338 | 127.6466056 | 6 |
| 1.8 | p | 0 | 0 | 1126.351501 | 118.6747688 | 6 |
| 1.8 | t | 0 | 0 | 1021.159359 | 13.48262695 | 14 |
| 1.8 | h | 0 | 0 | 1222.715659 | 215.038926 | 30 |
| 1.8 | lq | 0 | 0 | 1097.236602 | 89.55986979 | 7 |
| 1.8 | lp | 0 | 0 | 1122.466659 | 114.7899263 | 6 |
| 1.8 | lt | 0 | 0.111111111 | 1028.176774 | 20.50004161 | 15 |
| 1.8 | lh | 0 | 0 | 1058.094734 | 50.41800158 | 12 |
| 1.8 | qp | 0 | 0 | 1099.081055 | 91.40432292 | 7 |
| 1.8 | qt | 0 | 0.111111111 | 1019.27441 | 11.59767703 | 13 |
| 1.8 | qh | 0 | 0 | 1073.596699 | 65.91996659 | 15 |
| 1.8 | pt | 0 | 0.222222222 | 1026.715623 | 19.03889095 | 15 |
| 1.8 | ph | 0 | 0 | 1095.066018 | 87.38928528 | 18 |
| 1.8 | th | 0 | 0 | 1023.217574 | 15.54084121 | 15 |
| 1.8 | lqp | 0 | 0 | 1098.794085 | 91.11735291 | 7 |
| 1.8 | lqt | 0 | 0.111111111 | 1028.372746 | 20.69601358 | 15 |
| 1.8 | lqh | 0 | 0 | 1055.929594 | 48.25286175 | 12 |
| 1.8 | lpt | 0 | 0.111111111 | 1033.015604 | 25.33887165 | 16 |
| 1.8 | lph | 0 | 0 | 1060.233072 | 52.55633907 | 12 |
| 1.8 | qpt | 0 | 0.111111111 | 1032.91851 | 25.24177765 | 16 |
| 1.8 | qph | 0 | 0 | 1063.179839 | 55.50310692 | 13 |
| 1.8 | qth | 0 | 0.111111111 | 1024.030809 | 16.35407674 | 15 |
| 1.8 | pth | 0 | 0.111111111 | 1035.558798 | 27.88206548 | 17 |
| 1.8 | lqpt | 0 | 0.111111111 | 1032.864948 | 25.18821558 | 16 |
| 1.8 | lqph | 0 | 0 | 1067.602765 | 59.92603232 | 14 |
| 1.8 | lqth | 0 | 0.111111111 | 1039.745581 | 32.06884869 | 18 |
| 1.8 | lpth | 0 | 0.111111111 | 1047.679163 | 40.00243088 | 19 |
| 1.8 | lqpth | 0 | 0.111111111 | 1041.82138 | 34.14464756 | 18 |
| 1.9 | l | 0 | 0 | 1131.191613 | 123.5148803 | 6 |
| 1.9 | q | 0 | 0 | 1137.150721 | 129.4739886 | 6 |
| 1.9 | p | 0 | 0 | 1127.215512 | 119.5387796 | 6 |
| 1.9 | t | 0 | 0 | 1019.970176 | 12.29344375 | 13 |
| 1.9 | h | 0 | 0 | 1269.618616 | 261.941884 | 32 |
| 1.9 | lq | 0 | 0 | 1099.58368 | 91.9069475 | 7 |
| 1.9 | lp | 0 | 0 | 1122.837197 | 115.160465 | 6 |
| 1.9 | lt | 0 | 0.111111111 | 1026.447936 | 18.77120331 | 14 |
| 1.9 | lh | 0 | 0 | 1061.159284 | 53.48255197 | 12 |
| 1.9 | qp | 0 | 0 | 1101.345167 | 93.66843478 | 7 |
| 1.9 | qt | 0 | 0.111111111 | 1022.484085 | 14.80735214 | 13 |
| 1.9 | qh | 0 | 0 | 1065.15732 | 57.48058712 | 12 |
| 1.9 | pt | 0 | 0.111111111 | 1028.999867 | 21.32313468 | 15 |
| 1.9 | ph | 0 | 0 | 1088.651494 | 80.97476174 | 16 |
| 1.9 | th | 0 | 0 | 1025.55201 | 17.8752777 | 15 |
| 1.9 | lqp | 0 | 0 | 1101.049134 | 93.37240104 | 7 |
| 1.9 | lqt | 0 | 0.111111111 | 1031.431586 | 23.75485346 | 15 |
| 1.9 | lqh | 0 | 0 | 1067.54598 | 59.86924761 | 14 |
| 1.9 | lpt | 0 | 0.111111111 | 1035.540433 | 27.86370011 | 16 |
| 1.9 | lph | 0 | 0 | 1059.912982 | 52.23624934 | 11 |
| 1.9 | qpt | 0 | 0.111111111 | 1030.599185 | 22.92245298 | 15 |
| 1.9 | qph | 0 | 0 | 1059.471821 | 51.79508826 | 11 |
| 1.9 | qth | 0 | 0.111111111 | 1026.716501 | 19.03976846 | 15 |
| 1.9 | pth | 0 | 0.111111111 | 1033.232811 | 25.55607856 | 16 |
| 1.9 | lqpt | 0 | 0.111111111 | 1035.496054 | 27.81932131 | 16 |
| 1.9 | lqph | 0 | 0 | 1067.080779 | 59.40404652 | 13 |
| 1.9 | lqth | 0 | 0.111111111 | 1037.028895 | 29.35216202 | 17 |
| 1.9 | lpth | 0 | 0.111111111 | 1056.890115 | 49.21338205 | 20 |
| 1.9 | lqpth | 0 | 0.111111111 | 1057.087809 | 49.41107699 | 20 |
| 2 | l | 0 | 0 | 1131.694666 | 124.0179337 | 6 |
| 2.0 | q | 0 | 0 | 1138.955428 | 131.2786954 | 6 |
| 2.0 | p | 0 | 0 | 1128.051608 | 120.3748751 | 6 |
| 2.0 | t | 0 | 0 | 1018.718767 | 11.04203485 | 12 |
| 2.0 | h | 0 | 0 | 1415.830442 | 408.153709 | 36 |
| 2.0 | lq | 0 | 0 | 1101.836955 | 94.16022228 | 7 |
| 2.0 | lp | 0 | 0 | 1123.217102 | 115.5403699 | 6 |
| 2.0 | lt | 0 | 0.111111111 | 1028.906356 | 21.22962335 | 14 |
| 2.0 | lh | 0 | 0 | 1061.700405 | 54.02367229 | 11 |
| 2.0 | qp | 0 | 0 | 1103.571025 | 95.89429224 | 7 |
| 2.0 | qt | 0 | 0.111111111 | 1025.785401 | 18.10866821 | 13 |
| 2.0 | qh | 0 | 0 | 1061.395615 | 53.71888203 | 10 |
| 2.0 | pt | 0 | 0.111111111 | 1031.470245 | 23.79351249 | 15 |
| 2.0 | ph | 0 | 0 | 1103.672379 | 95.99564605 | 18 |
| 2.0 | th | 0 | 0 | 1032.595319 | 24.91858611 | 16 |
| 2.0 | lqp | 0 | 0 | 1103.11222 | 95.43548705 | 7 |
| 2.0 | lqt | 0 | 0.111111111 | 1029.632894 | 21.9561615 | 14 |
| 2.0 | lqh | 0 | 0 | 1056.134973 | 48.45824074 | 10 |
| 2.0 | lpt | 0 | 0.111111111 | 1038.139032 | 30.4622991 | 16 |
| 2.0 | lph | 0 | 0 | 1067.73227 | 60.05553715 | 12 |
| 2.0 | qpt | 0 | 0.111111111 | 1033.214466 | 25.53773333 | 15 |
| 2.0 | qph | 0 | 0 | 1066.927333 | 59.25060006 | 12 |
| 2.0 | qth | 0 | 0.111111111 | 1024.925141 | 17.24840863 | 14 |
| 2.0 | pth | 0 | 0.111111111 | 1035.815188 | 28.13845549 | 16 |
| 2.0 | lqpt | 0 | 0.111111111 | 1033.298037 | 25.62130417 | 15 |
| 2.0 | lqph | 0 | 0 | 1066.339133 | 58.66240095 | 12 |
| 2.0 | lqth | 0 | 0.111111111 | 1034.542806 | 26.86607379 | 16 |
| 2.0 | lpth | 0 | 0.111111111 | 1047.216922 | 39.54018909 | 18 |
| 2.0 | lqpth | 0 | 0.111111111 | 1053.368474 | 45.69174119 | 19 |
| 2.1 | l | 0 | 0 | 1132.197801 | 124.5210686 | 6 |
| 2.1 | q | 0 | 0 | 1140.704006 | 133.0272736 | 6 |
| 2.1 | p | 0 | 0 | 1128.800217 | 121.1234847 | 6 |
| 2.1 | t | 0 | 0 | 1017.222606 | 9.545873061 | 11 |
| 2.1 | h | 0 | 0 | 1787.907294 | 780.2305615 | 40 |
| 2.1 | lq | 0 | 0 | 1103.916106 | 96.23937375 | 7 |
| 2.1 | lp | 0 | 0 | 1123.61709 | 115.9403577 | 6 |
| 2.1 | lt | 0 | 0.111111111 | 1027.167453 | 19.49072038 | 13 |
| 2.1 | lh | 0 | 0 | 1057.93055 | 50.25381796 | 9 |
| 2.1 | qp | 0 | 0 | 1105.701312 | 98.02457917 | 7 |
| 2.1 | qt | 0 | 0.111111111 | 1029.212891 | 21.53615841 | 13 |
| 2.1 | qh | 0 | 0 | 1064.761832 | 57.08509904 | 10 |
| 2.1 | pt | 0 | 0.111111111 | 1029.439675 | 21.7629422 | 14 |
| 2.1 | ph | 0 | 0 | 1096.603925 | 88.92719218 | 16 |
| 2.1 | th | 0 | 0 | 1034.978027 | 27.30129444 | 16 |
| 2.1 | lqp | 0 | 0 | 1105.322318 | 97.64558567 | 7 |
| 2.1 | lqt | 0 | 0.111111111 | 1027.777772 | 20.10103994 | 13 |
| 2.1 | lqh | 0 | 0 | 1059.770169 | 52.09343686 | 10 |
| 2.1 | lpt | 0 | 0.111111111 | 1040.805205 | 33.12847286 | 16 |
| 2.1 | lph | 0 | 0 | 1067.129421 | 59.45268801 | 11 |
| 2.1 | qpt | 0 | 0.111111111 | 1035.758917 | 28.08218455 | 15 |
| 2.1 | qph | 0 | 0 | 1066.747201 | 59.07046857 | 11 |
| 2.1 | qth | 0 | 0.111111111 | 1027.631082 | 19.95434979 | 14 |
| 2.1 | pth | 0 | 0.111111111 | 1033.699777 | 26.02304483 | 15 |
| 2.1 | lqpt | 0 | 0.111111111 | 1035.892313 | 28.21558048 | 15 |
| 2.1 | lqph | 0 | 0 | 1070.213254 | 62.53652161 | 12 |
| 2.1 | lqth | 0 | 0.111111111 | 1032.186834 | 24.51010122 | 15 |
| 2.1 | lpth | 0 | 0.111111111 | 1044.645926 | 36.96919366 | 17 |
| 2.1 | lqpth | 0 | 0.111111111 | 1034.905958 | 27.22922513 | 15 |
| 2.2 | l | 0 | 0 | 1132.71962 | 125.0428872 | 6 |
| 2.2 | q | 0 | 0 | 1142.434344 | 134.7576117 | 6 |
| 2.2 | p | 0 | 0 | 1129.453508 | 121.7767752 | 6 |
| 2.2 | t | 0 | 0 | 1019.378809 | 11.70207679 | 11 |
| 2.2 | h | 0 | 0 | 1308.788637 | 301.1119046 | 33 |
| 2.2 | lq | 0 | 0 | 1105.999753 | 98.32302075 | 7 |
| 2.2 | lp | 0 | 0 | 1124.017312 | 116.3405796 | 6 |
| 2.2 | lt | 0 | 0.111111111 | 1029.373917 | 21.69718426 | 13 |
| 2.2 | lh | 0 | 0 | 1061.147855 | 53.47112246 | 9 |
| 2.2 | qp | 0 | 0 | 1107.772574 | 100.0958416 | 7 |
| 2.2 | qt | 0 | 0.111111111 | 1028.440404 | 20.76367184 | 12 |
| 2.2 | qh | 0 | 0 | 1071.492863 | 63.81613049 | 11 |
| 2.2 | pt | 0 | 0.111111111 | 1031.759837 | 24.08310418 | 14 |
| 2.2 | ph | 0 | 0 | 1100.387304 | 92.71057117 | 16 |
| 2.2 | th | 0 | 0 | 1032.535271 | 24.85853891 | 15 |
| 2.2 | lqp | 0 | 0 | 1107.36543 | 99.68869747 | 7 |
| 2.2 | lqt | 0 | 0.111111111 | 1029.829468 | 22.15273502 | 13 |
| 2.2 | lqh | 0 | 0 | 1066.494835 | 58.81810217 | 11 |
| 2.2 | lpt | 0 | 0.111111111 | 1038.679574 | 31.00284178 | 15 |
| 2.2 | lph | 0 | 0 | 1078.190741 | 70.51400831 | 13 |
| 2.2 | qpt | 0 | 0.111111111 | 1033.941609 | 26.26487697 | 14 |
| 2.2 | qph | 0 | 0 | 1074.11378 | 66.43704771 | 12 |
| 2.2 | qth | 0 | 0.111111111 | 1025.715803 | 18.0390702 | 13 |
| 2.2 | pth | 0 | 0.111111111 | 1036.262745 | 28.58601256 | 15 |
| 2.2 | lqpt | 0 | 0.111111111 | 1033.973456 | 26.29672336 | 14 |
| 2.2 | lqph | 0 | 0 | 1074.185101 | 66.50836894 | 12 |
| 2.2 | lqth | 0 | 0.111111111 | 1034.686567 | 27.00983496 | 15 |
| 2.2 | lpth | 0 | 0.111111111 | 1042.078504 | 34.40177118 | 16 |
| 2.2 | lqpth | 0 | 0.111111111 | 1042.34019 | 34.66345776 | 16 |
| 2.3 | l | 0 | 0 | 1133.260143 | 125.5834103 | 6 |
| 2.3 | q | 0 | 0 | 1144.124593 | 136.4478605 | 6 |
| 2.3 | p | 0 | 0 | 1130.091234 | 122.4145018 | 6 |
| 2.3 | t | 0 | 0 | 1021.552653 | 13.87592051 | 11 |
| 2.3 | h | 0 | 0 | 5107.806114 | 4100.129381 | 44 |
| 2.3 | lq | 0 | 0 | 1107.993235 | 100.3165023 | 7 |
| 2.3 | lp | 0 | 0 | 1124.425249 | 116.7485167 | 6 |
| 2.3 | lt | 0 | 0.111111111 | 1031.594413 | 23.91768092 | 13 |
| 2.3 | lh | 0 | 0 | 1068.584396 | 60.90766337 | 10 |
| 2.3 | qp | 0 | 0 | 1109.707832 | 102.0310997 | 7 |
| 2.3 | qt | 0 | 0.111111111 | 1026.978786 | 19.30205336 | 11 |
| 2.3 | qh | 0 | 0 | 1071.629057 | 63.95232483 | 10 |
| 2.3 | pt | 0 | 0.111111111 | 1034.290253 | 26.61352028 | 14 |
| 2.3 | ph | 0 | 0 | 1099.140488 | 91.46375523 | 15 |
| 2.3 | th | 0 | 0 | 1034.921497 | 27.2447649 | 15 |
| 2.3 | lqp | 0 | 0 | 1109.33492 | 101.6581874 | 7 |
| 2.3 | lqt | 0 | 0.111111111 | 1031.96256 | 24.28582759 | 13 |
| 2.3 | lqh | 0 | 0 | 1066.507226 | 58.83049308 | 10 |
| 2.3 | lpt | 0 | 0.111111111 | 1040.902401 | 33.22566827 | 15 |
| 2.3 | lph | 0 | 0 | 1074.271454 | 66.59472192 | 11 |
| 2.3 | qpt | 0 | 0.111111111 | 1036.050562 | 28.37382998 | 14 |
| 2.3 | qph | 0 | 0 | 1070.339519 | 62.6627869 | 10 |
| 2.3 | qth | 0 | 0.111111111 | 1028.157911 | 20.48117886 | 13 |
| 2.3 | pth | 0 | 0.111111111 | 1034.285106 | 26.60837336 | 14 |
| 2.3 | lqpt | 0 | 0.111111111 | 1036.095914 | 28.41918187 | 14 |
| 2.3 | lqph | 0 | 0 | 1077.146471 | 69.46973824 | 12 |
| 2.3 | lqth | 0 | 0.111111111 | 1037.068153 | 29.39142098 | 15 |
| 2.3 | lpth | 0 | 0.111111111 | 1044.52134 | 36.84460729 | 16 |
| 2.3 | lqpth | 0 | 0.111111111 | 1040.001836 | 32.32510328 | 15 |
| 2.4 | l | 0 | 0 | 1133.818589 | 126.1418565 | 6 |
| 2.4 | q | 0 | 0 | 1145.78775 | 138.111018 | 6 |
| 2.4 | p | 0 | 0 | 1130.707362 | 123.0306293 | 6 |
| 2.4 | t | 0 | 0 | 1023.770262 | 16.09352904 | 11 |
| 2.4 | h | 0 | 0 | 1385.712563 | 378.0358305 | 35 |
| 2.4 | lq | 0 | 0 | 1109.901997 | 102.2252649 | 7 |
| 2.4 | lp | 0 | 0 | 1124.879338 | 117.2026059 | 6 |
| 2.4 | lt | 0 | 0.111111111 | 1033.724509 | 26.04777681 | 13 |
| 2.4 | lh | 0 | 0 | 1071.650782 | 63.97404996 | 10 |
| 2.4 | qp | 0 | 0 | 1111.629314 | 103.9525811 | 7 |
| 2.4 | qt | 0 | 0.111111111 | 1029.026786 | 21.35005377 | 11 |
| 2.4 | qh | 0 | 0 | 1074.517186 | 66.84045315 | 10 |
| 2.4 | pt | 0 | 0.111111111 | 1036.741926 | 29.06519344 | 14 |
| 2.4 | ph | 0 | 0 | 1098.625412 | 90.94867915 | 14 |
| 2.4 | th | 0 | 0 | 1028.507077 | 20.83034441 | 13 |
| 2.4 | lqp | 0 | 0 | 1111.263708 | 103.586975 | 7 |
| 2.4 | lqt | 0 | 0.111111111 | 1034.085001 | 26.40826893 | 13 |
| 2.4 | lqh | 0 | 0 | 1069.246892 | 61.57015942 | 10 |
| 2.4 | lpt | 0 | 0.111111111 | 1043.223944 | 35.54721139 | 15 |
| 2.4 | lph | 0 | 0 | 1073.360688 | 65.68395596 | 10 |
| 2.4 | qpt | 0 | 0.111111111 | 1038.297266 | 30.62053377 | 14 |
| 2.4 | qph | 0 | 0 | 1076.5585 | 68.88176783 | 11 |
| 2.4 | qth | 0 | 0.111111111 | 1030.565035 | 22.8883023 | 13 |
| 2.4 | pth | 0 | 0.111111111 | 1036.718657 | 29.04192403 | 14 |
| 2.4 | lqpt | 0 | 0.111111111 | 1038.258475 | 30.58174218 | 14 |
| 2.4 | lqph | 0 | 0 | 1087.776191 | 80.09945858 | 14 |
| 2.4 | lqth | 0 | 0 | 1049.313033 | 41.6363004 | 17 |
| 2.4 | lpth | 0 | 0.111111111 | 1047.186376 | 39.509643 | 16 |
| 2.4 | lqpth | 0 | 0.111111111 | 1042.357757 | 34.68102483 | 15 |
| 2.5 | l | 0 | 0 | 1134.393408 | 126.7166756 | 6 |
| 2.5 | q | 0 | 0 | 1147.424377 | 139.7476445 | 6 |
| 2.5 | p | 0 | 0 | 1131.282155 | 123.6054227 | 6 |
| 2.5 | t | 0 | 0 | 1022.458594 | 14.78186176 | 10 |
| 2.5 | h | 0 | 0 | 5115.361552 | 4107.68482 | 44 |
| 2.5 | lq | 0 | 0 | 1115.601031 | 107.9242986 | 8 |
| 2.5 | lp | 0 | 0 | 1125.291597 | 117.6148644 | 6 |
| 2.5 | lt | 0 | 0.111111111 | 1035.984095 | 28.3073623 | 13 |
| 2.5 | lh | 0 | 0 | 1071.661416 | 63.98468392 | 9 |
| 2.5 | qp | 0 | 0 | 1113.49867 | 105.8219379 | 7 |
| 2.5 | qt | 0 | 0.111111111 | 1031.069938 | 23.39320558 | 11 |
| 2.5 | qh | 0 | 0 | 1078.105259 | 70.42852605 | 10 |
| 2.5 | pt | 0 | 0.111111111 | 1039.314625 | 31.63789292 | 14 |
| 2.5 | ph | 0 | 0 | 1102.104335 | 94.42760291 | 14 |
| 2.5 | th | 0 | 0 | 1034.912611 | 27.23587851 | 14 |
| 2.5 | lqp | 0 | 0 | 1113.09186 | 105.4151273 | 7 |
| 2.5 | lqt | 0 | 0.111111111 | 1036.311106 | 28.63437302 | 13 |
| 2.5 | lqh | 0 | 0 | 1068.896334 | 61.2196019 | 9 |
| 2.5 | lpt | 0 | 0.111111111 | 1045.644231 | 37.96749831 | 15 |
| 2.5 | lph | 0 | 0 | 1079.918109 | 72.24137657 | 11 |
| 2.5 | qpt | 0 | 0.111111111 | 1040.688261 | 33.01152875 | 14 |
| 2.5 | qph | 0 | 0 | 1079.867099 | 72.19036604 | 11 |
| 2.5 | qth | 0 | 0.111111111 | 1032.951642 | 25.27490977 | 13 |
| 2.5 | pth | 0 | 0.111111111 | 1039.211907 | 31.53517457 | 14 |
| 2.5 | lqpt | 0 | 0.111111111 | 1040.6126 | 32.93586797 | 14 |
| 2.5 | lqph | 0 | 0 | 1079.514197 | 71.83746487 | 11 |
| 2.5 | lqth | 0 | 0.111111111 | 1046.73274 | 39.056007 | 16 |
| 2.5 | lpth | 0 | 0.111111111 | 1054.910595 | 47.23386294 | 17 |
| 2.5 | lqpth | 0 | 0.111111111 | 1044.875086 | 37.19835385 | 15 |
| 2.6 | l | 0 | 0 | 1134.97177 | 127.2950378 | 6 |
| 2.6 | q | 0 | 0 | 1149.033372 | 141.3566394 | 6 |
| 2.6 | p | 0 | 0 | 1131.856092 | 124.1793599 | 6 |
| 2.6 | t | 0 | 0 | 1024.549802 | 16.87306917 | 10 |
| 2.6 | h | 0 | 0 | 1355.593423 | 347.9166909 | 34 |
| 2.6 | lq | 0 | 0 | 1118.520524 | 110.8437919 | 8 |
| 2.6 | lp | 0 | 0 | 1125.756868 | 118.0801359 | 6 |
| 2.6 | lt | 0 | 0.111111111 | 1038.251273 | 30.57454055 | 13 |
| 2.6 | lh | 0 | 0 | 1077.412383 | 69.73565066 | 10 |
| 2.6 | qp | 0 | 0 | 1112.45558 | 104.7788472 | 6 |
| 2.6 | qt | 0 | 0.111111111 | 1033.130587 | 25.45385488 | 11 |
| 2.6 | qh | 0 | 0 | 1078.194995 | 70.51826251 | 9 |
| 2.6 | pt | 0 | 0.111111111 | 1041.798622 | 34.1218892 | 14 |
| 2.6 | ph | 0 | 0 | 1120.525914 | 112.8491812 | 17 |
| 2.6 | th | 0 | 0 | 1037.220334 | 29.54360178 | 14 |
| 2.6 | lqp | 0 | 0 | 1114.853543 | 107.1768108 | 7 |
| 2.6 | lqt | 0 | 0.111111111 | 1038.548981 | 30.87224867 | 13 |
| 2.6 | lqh | 0 | 0 | 1071.995019 | 64.31828634 | 9 |
| 2.6 | lpt | 0 | 0.111111111 | 1048.197831 | 40.52109845 | 15 |
| 2.6 | lph | 0 | 0 | 1079.341281 | 71.66454847 | 10 |
| 2.6 | qpt | 0 | 0.111111111 | 1043.020479 | 35.34374646 | 14 |
| 2.6 | qph | 0 | 0 | 1086.599949 | 78.92321694 | 12 |
| 2.6 | qth | 0 | 0.111111111 | 1035.348826 | 27.67209398 | 13 |
| 2.6 | pth | 0 | 0.111111111 | 1041.802167 | 34.12543417 | 14 |
| 2.6 | lqpt | 0 | 0.111111111 | 1047.431527 | 39.75479428 | 15 |
| 2.6 | lqph | 0 | 0 | 1086.099919 | 78.42318629 | 12 |
| 2.6 | lqth | 0 | 0.111111111 | 1049.280053 | 41.60332069 | 16 |
| 2.6 | lpth | 0 | 0.111111111 | 1052.218856 | 44.54212348 | 16 |
| 2.6 | lqpth | 0 | 0.111111111 | 1052.082515 | 44.40578259 | 16 |
| 2.7 | l | 0 | 0 | 1135.555711 | 127.8789788 | 6 |
| 2.7 | q | 0 | 0 | 1150.609978 | 142.9332454 | 6 |
| 2.7 | p | 0 | 0 | 1132.420632 | 124.7438995 | 6 |
| 2.7 | t | 0 | 0 | 1026.653802 | 18.9770695 | 10 |
| 2.7 | h | 0 | 0 | 1241.675437 | 233.9987042 | 29 |
| 2.7 | lq | 0 | 0 | 1117.301956 | 109.6252238 | 7 |
| 2.7 | lp | 0 | 0 | 1126.215603 | 118.5388708 | 6 |
| 2.7 | lt | 0 | 0.111111111 | 1040.529477 | 32.85274494 | 13 |
| 2.7 | lh | 0 | 0 | 1077.10021 | 69.42347777 | 9 |
| 2.7 | qp | 0 | 0 | 1114.128293 | 106.4515609 | 6 |
| 2.7 | qt | 0 | 0.111111111 | 1035.213412 | 27.5366799 | 11 |
| 2.7 | qh | 0 | 0 | 1080.718516 | 73.04178315 | 9 |
| 2.7 | pt | 0 | 0.111111111 | 1044.331553 | 36.65482042 | 14 |
| 2.7 | ph | 0 | 0 | 1113.545814 | 105.869081 | 15 |
| 2.7 | th | 0 | 0 | 1035.305391 | 27.62865852 | 13 |
| 2.7 | lqp | 0 | 0 | 1113.747795 | 106.0710622 | 6 |
| 2.7 | lqt | 0 | 0.111111111 | 1040.707374 | 33.03064197 | 13 |
| 2.7 | lqh | 0 | 0 | 1074.34774 | 66.67100785 | 9 |
| 2.7 | lpt | 0 | 0.111111111 | 1045.944315 | 38.26758269 | 14 |
| 2.7 | lph | 0 | 0 | 1082.373563 | 74.69683036 | 10 |
| 2.7 | qpt | 0 | 0.111111111 | 1045.290062 | 37.61332975 | 14 |
| 2.7 | qph | 0 | 0 | 1082.140536 | 74.46380395 | 10 |
| 2.7 | qth | 0 | 0.111111111 | 1041.939637 | 34.26290472 | 14 |
| 2.7 | pth | 0 | 0.111111111 | 1044.303213 | 36.62648015 | 14 |
| 2.7 | lqpt | 0 | 0.111111111 | 1049.870022 | 42.19328947 | 15 |
| 2.7 | lqph | 0 | 0 | 1089.122945 | 81.44621248 | 12 |
| 2.7 | lqth | 0 | 0 | 1051.709861 | 44.03312846 | 16 |
| 2.7 | lpth | 0 | 0.111111111 | 1050.049475 | 42.37274216 | 15 |
| 2.7 | lqpth | 0 | 0.111111111 | 1049.619402 | 41.94266901 | 15 |
| 2.8 | l | 0 | 0 | 1136.15975 | 128.4830171 | 6 |
| 2.8 | q | 0 | 0 | 1152.161746 | 144.4850132 | 6 |
| 2.8 | p | 0 | 0 | 1132.991332 | 125.3145995 | 6 |
| 2.8 | t | 0 | 0 | 1028.796392 | 21.11965982 | 10 |
| 2.8 | h | 0 | 0 | 1262.681491 | 255.0047589 | 30 |
| 2.8 | lq | 0 | 0 | 1118.963937 | 111.2872047 | 7 |
| 2.8 | lp | 0 | 0 | 1126.721648 | 119.044915 | 6 |
| 2.8 | lt | 0 | 0.111111111 | 1042.909272 | 35.23253924 | 13 |
| 2.8 | lh | 0 | 0 | 1083.2026 | 75.5258675 | 10 |
| 2.8 | qp | 0 | 0 | 1115.771578 | 108.0948453 | 6 |
| 2.8 | qt | 0 | 0.111111111 | 1037.292926 | 29.61619329 | 11 |
| 2.8 | qh | 0 | 0 | 1083.725772 | 76.0490395 | 9 |
| 2.8 | pt | 0 | 0.111111111 | 1046.97337 | 39.29663774 | 14 |
| 2.8 | ph | 0 | 0 | 1108.660125 | 100.9833926 | 13 |
| 2.8 | th | 0 | 0 | 1037.594892 | 29.91815989 | 13 |
| 2.8 | lqp | 0 | 0 | 1115.372175 | 107.6954427 | 6 |
| 2.8 | lqt | 0 | 0.111111111 | 1047.201025 | 39.52429268 | 14 |
| 2.8 | lqh | 0 | 0 | 1077.38577 | 69.70903781 | 9 |
| 2.8 | lpt | 0 | 0.111111111 | 1048.532627 | 40.85589459 | 14 |
| 2.8 | lph | 0 | 0 | 1089.103654 | 81.42692172 | 11 |
| 2.8 | qpt | 0 | 0.111111111 | 1047.626327 | 39.94959407 | 14 |
| 2.8 | qph | 0 | 0 | 1085.284881 | 77.60814868 | 10 |
| 2.8 | qth | 0 | 0.111111111 | 1044.513994 | 36.83726143 | 14 |
| 2.8 | pth | 0 | 0.111111111 | 1046.961299 | 39.28456601 | 14 |
| 2.8 | lqpt | 0 | 0.111111111 | 1052.191792 | 44.51505924 | 15 |
| 2.8 | lqph | 0 | 0 | 1092.508129 | 84.83139606 | 12 |
| 2.8 | lqth | 0 | 0 | 1054.307919 | 46.63118697 | 16 |
| 2.8 | lpth | 0 | 0.111111111 | 1048.07165 | 40.39491726 | 14 |
| 2.8 | lqpth | 0 | 0.111111111 | 1052.019925 | 44.34319292 | 15 |
| 2.9 | l | 0 | 0 | 1136.773769 | 129.0970362 | 6 |
| 2.9 | q | 0 | 0 | 1153.702576 | 146.0258436 | 6 |
| 2.9 | p | 0 | 0 | 1133.557999 | 125.8812668 | 6 |
| 2.9 | t | 0 | 0 | 1030.953147 | 23.27641432 | 10 |
| 2.9 | h | 0 | 0 | 1507.91372 | 500.2369871 | 37 |
| 2.9 | lq | 0 | 0 | 1120.592202 | 112.9154692 | 7 |
| 2.9 | lp | 0 | 0 | 1127.172117 | 119.495385 | 6 |
| 2.9 | lt | 0 | 0.111111111 | 1045.187827 | 37.51109475 | 13 |
| 2.9 | lh | 0 | 0 | 1082.61323 | 74.93649763 | 9 |
| 2.9 | qp | 0 | 0 | 1117.328629 | 109.6518969 | 6 |
| 2.9 | qt | 0 | 0.111111111 | 1039.411687 | 31.73495482 | 11 |
| 2.9 | qh | 0 | 0 | 1086.518496 | 78.84176392 | 9 |
| 2.9 | pt | 0 | 0.111111111 | 1049.544624 | 41.86789132 | 14 |
| 2.9 | ph | 0 | 0 | 1103.9026 | 96.22586713 | 11 |
| 2.9 | th | 0 | 0 | 1039.958222 | 32.28148969 | 13 |
| 2.9 | lqp | 0 | 0 | 1116.888241 | 109.2115081 | 6 |
| 2.9 | lqt | 0 | 0.111111111 | 1045.420934 | 37.74420125 | 13 |
| 2.9 | lqh | 0 | 0 | 1076.830004 | 69.15327196 | 8 |
| 2.9 | lpt | 0 | 0.111111111 | 1051.100396 | 43.42366341 | 14 |
| 2.9 | lph | 0 | 0 | 1084.691285 | 77.0145522 | 9 |
| 2.9 | qpt | 0 | 0.111111111 | 1050.11305 | 42.43631736 | 14 |
| 2.9 | qph | 0 | 0 | 1084.55674 | 76.88000772 | 9 |
| 2.9 | qth | 0 | 0.111111111 | 1047.017615 | 39.34088225 | 14 |
| 2.9 | pth | 0 | 0.111111111 | 1049.500536 | 41.82380337 | 14 |
| 2.9 | lqpt | 0 | 0.111111111 | 1054.775451 | 47.09871807 | 15 |
| 2.9 | lqph | 0 | 0 | 1095.172597 | 87.49586412 | 12 |
| 2.9 | lqth | 0 | 0 | 1056.836862 | 49.16012966 | 16 |
| 2.9 | lpth | 0 | 0.111111111 | 1055.000752 | 47.32401965 | 15 |
| 2.9 | lqpth | 0 | 0.111111111 | 1054.860248 | 47.18351512 | 15 |
| 3 | l | 0 | 0 | 1137.404501 | 129.727769 | 6 |
| 3.0 | q | 0 | 0 | 1155.216715 | 147.539982 | 6 |
| 3.0 | p | 0 | 0 | 1134.118807 | 126.4420741 | 6 |
| 3.0 | t | 0 | 0 | 1033.136668 | 25.45993504 | 10 |
| 3.0 | h | 0 | 0 | 1822.200032 | 814.5232994 | 40 |
| 3.0 | lq | 0 | 0 | 1122.173817 | 114.4970846 | 7 |
| 3.0 | lp | 0 | 0 | 1127.706573 | 120.0298403 | 6 |
| 3.0 | lt | 0 | 0.111111111 | 1047.540919 | 39.86418628 | 13 |
| 3.0 | lh | 0 | 0 | 1082.047659 | 74.3709268 | 8 |
| 3.0 | qp | 0 | 0 | 1118.762438 | 111.0857052 | 6 |
| 3.0 | qt | 0 | 0.111111111 | 1041.564143 | 33.8874101 | 11 |
| 3.0 | qh | 0 | 0 | 1089.205486 | 81.52875368 | 9 |
| 3.0 | pt | 0 | 0.111111111 | 1047.723089 | 40.04635648 | 13 |
| 3.0 | ph | 0 | 0 | 1106.796438 | 99.11970566 | 11 |
| 3.0 | th | 0 | 0 | 1042.270781 | 34.59404867 | 13 |
| 3.0 | lqp | 0 | 0 | 1118.404625 | 110.7278927 | 6 |
| 3.0 | lqt | 0 | 0.111111111 | 1051.940155 | 44.26342223 | 14 |
| 3.0 | lqh | 0 | 0 | 1081.589747 | 73.91301409 | 9 |
| 3.0 | lpt | 0 | 0.111111111 | 1053.348951 | 45.67221876 | 14 |
| 3.0 | lph | 0 | 0 | 1083.986221 | 76.30948881 | 8 |
| 3.0 | qpt | 0 | 0.111111111 | 1052.56087 | 44.88413723 | 14 |
| 3.0 | qph | 0 | 0 | 1083.968625 | 76.29189235 | 8 |
| 3.0 | qth | 0 | 0.111111111 | 1049.563023 | 41.88629054 | 14 |
| 3.0 | pth | 0 | 0.111111111 | 1047.73648 | 40.05974797 | 13 |
| 3.0 | lqpt | 0 | 0.111111111 | 1057.058166 | 49.3814335 | 15 |
| 3.0 | lqph | 0 | 0 | 1087.081086 | 79.40435335 | 9 |
| 3.0 | lqth | 0 | 0 | 1059.304071 | 51.62733861 | 16 |
| 3.0 | lpth | 0 | 0.111111111 | 1053.088186 | 45.41145381 | 14 |
| 3.0 | lqpth | 0 | 0.111111111 | 1057.026071 | 49.3493384 | 15 |
| 3.1 | l | 0 | 0 | 1138.031132 | 130.3543994 | 6 |
| 3.1 | q | 0 | 0 | 1156.72002 | 149.0432872 | 6 |
| 3.1 | p | 0 | 0 | 1134.674349 | 126.9976161 | 6 |
| 3.1 | t | 0 | 0 | 1035.328599 | 27.65186694 | 10 |
| 3.1 | h | 0 | 0 | 1411.994287 | 404.3175542 | 35 |
| 3.1 | lq | 0 | 0 | 1123.688129 | 116.0113967 | 7 |
| 3.1 | lp | 0 | 0 | 1128.212047 | 120.5353142 | 6 |
| 3.1 | lt | 0 | 0.111111111 | 1049.93305 | 42.25631735 | 13 |
| 3.1 | lh | 0 | 0 | 1081.002178 | 73.3254452 | 7 |
| 3.1 | qp | 0 | 0 | 1120.244403 | 112.5676701 | 6 |
| 3.1 | qt | 0 | 0.111111111 | 1047.502691 | 39.82595879 | 12 |
| 3.1 | qh | 0 | 0 | 1085.462711 | 77.78597818 | 7 |
| 3.1 | pt | 0 | 0.111111111 | 1050.259401 | 42.5826682 | 13 |
| 3.1 | ph | 0 | 0 | 1109.579209 | 101.902476 | 11 |
| 3.1 | th | 0 | 0 | 1048.847897 | 41.17116485 | 14 |
| 3.1 | lqp | 0 | 0 | 1119.836367 | 112.1596342 | 6 |
| 3.1 | lqt | 0 | 0.111111111 | 1050.231338 | 42.55460507 | 13 |
| 3.1 | lqh | 0 | 0 | 1083.796314 | 76.11958138 | 9 |
| 3.1 | lpt | 0 | 0.111111111 | 1055.693323 | 48.01659035 | 14 |
| 3.1 | lph | 0 | 0 | 1086.221471 | 78.54473847 | 8 |
| 3.1 | qpt | 0 | 0.111111111 | 1055.134215 | 47.45748278 | 14 |
| 3.1 | qph | 0 | 0 | 1089.491448 | 81.814715 | 9 |
| 3.1 | qth | 0 | 0.111111111 | 1052.083474 | 44.40674149 | 14 |
| 3.1 | pth | 0 | 0.111111111 | 1050.187751 | 42.51101883 | 13 |
| 3.1 | lqpt | 0 | 0.111111111 | 1059.723148 | 52.04641526 | 15 |
| 3.1 | lqph | 0 | 0 | 1086.40889 | 78.73215716 | 8 |
| 3.1 | lqth | 0 | 0 | 1057.291487 | 49.61475411 | 15 |
| 3.1 | lpth | 0 | 0.111111111 | 1060.032772 | 52.35603911 | 15 |
| 3.1 | lqpth | 0 | 0.111111111 | 1059.799783 | 52.12305001 | 15 |
| 3.2 | l | 0 | 0 | 1138.6719 | 130.9951671 | 6 |
| 3.2 | q | 0 | 0 | 1158.192098 | 150.515365 | 6 |
| 3.2 | p | 0 | 0 | 1135.237521 | 127.5607886 | 6 |
| 3.2 | t | 0 | 0 | 1037.557252 | 29.88051906 | 10 |
| 3.2 | h | 0 | 0 | 1415.737714 | 408.0609816 | 35 |
| 3.2 | lq | 0 | 0 | 1125.222859 | 117.5461264 | 7 |
| 3.2 | lp | 0 | 0 | 1128.71345 | 121.0367179 | 6 |
| 3.2 | lt | 0 | 0.111111111 | 1052.450141 | 44.7734081 | 13 |
| 3.2 | lh | 0 | 0 | 1083.056713 | 75.37998014 | 7 |
| 3.2 | qp | 0 | 0 | 1121.645124 | 113.968391 | 6 |
| 3.2 | qt | 0 | 0.111111111 | 1049.735823 | 42.05909025 | 12 |
| 3.2 | qh | 0 | 0 | 1087.652793 | 79.97606065 | 7 |
| 3.2 | pt | 0 | 0.111111111 | 1052.692023 | 45.01529072 | 13 |
| 3.2 | ph | 0 | 0 | 1105.533203 | 97.85647088 | 9 |
| 3.2 | th | 0 | 0 | 1047.01498 | 39.33824739 | 13 |
| 3.2 | lqp | 0 | 0 | 1121.231874 | 113.5551418 | 6 |
| 3.2 | lqt | 0 | 0.111111111 | 1052.667714 | 44.9909817 | 13 |
| 3.2 | lqh | 0 | 0 | 1086.03034 | 78.35360709 | 9 |
| 3.2 | lpt | 0 | 0.111111111 | 1054.259739 | 46.58300686 | 13 |
| 3.2 | lph | 0 | 0 | 1088.451183 | 80.77445014 | 8 |
| 3.2 | qpt | 0 | 0.111111111 | 1057.50102 | 49.82428792 | 14 |
| 3.2 | qph | 0 | 0 | 1088.461554 | 80.78482161 | 8 |
| 3.2 | qth | 0 | 0.111111111 | 1054.658636 | 46.98190312 | 14 |
| 3.2 | pth | 0 | 0.111111111 | 1052.708618 | 45.03188529 | 13 |
| 3.2 | lqpt | 0 | 0.111111111 | 1062.337864 | 54.6611312 | 15 |
| 3.2 | lqph | 0 | 0 | 1088.779268 | 81.10253517 | 8 |
| 3.2 | lqth | 0 | 0 | 1064.595543 | 56.91881083 | 16 |
| 3.2 | lpth | 0 | 0.111111111 | 1058.201254 | 50.52452194 | 14 |
| 3.2 | lqpth | 0 | 0.111111111 | 1062.351917 | 54.67518466 | 15 |
| 3.3 | l | 0 | 0 | 1139.312541 | 131.635808 | 6 |
| 3.3 | q | 0 | 0 | 1159.653705 | 151.9769728 | 6 |
| 3.3 | p | 0 | 0 | 1135.79625 | 128.1195176 | 6 |
| 3.3 | t | 0 | 0 | 1039.816899 | 32.14016697 | 10 |
| 3.3 | h | 0 | 0 | 1301.277276 | 293.6005431 | 31 |
| 3.3 | lq | 0 | 0 | 1126.678723 | 119.0019906 | 7 |
| 3.3 | lp | 0 | 0 | 1129.19712 | 121.5203878 | 6 |
| 3.3 | lt | 0 | 0.111111111 | 1054.927396 | 47.25066354 | 13 |
| 3.3 | lh | 0 | 0 | 1087.949028 | 80.27229593 | 8 |
| 3.3 | qp | 0 | 0 | 1122.971211 | 115.2944785 | 6 |
| 3.3 | qt | 0 | 0.111111111 | 1052.048362 | 44.37162943 | 12 |
| 3.3 | qh | 0 | 0 | 1089.845697 | 82.1689644 | 7 |
| 3.3 | pt | 0 | 0.111111111 | 1055.000511 | 47.32377868 | 13 |
| 3.3 | ph | 0 | 0 | 1114.367011 | 106.6902788 | 11 |
| 3.3 | th | 0 | 0 | 1053.525282 | 45.84854985 | 14 |
| 3.3 | lqp | 0 | 0 | 1122.544629 | 114.8678966 | 6 |
| 3.3 | lqt | 0 | 0.111111111 | 1055.024134 | 47.34740115 | 13 |
| 3.3 | lqh | 0 | 0 | 1088.052673 | 80.37594027 | 9 |
| 3.3 | lpt | 0 | 0.111111111 | 1056.694873 | 49.01814017 | 13 |
| 3.3 | lph | 0 | 0 | 1090.771791 | 83.09505847 | 8 |
| 3.3 | qpt | 0 | 0.111111111 | 1059.889892 | 52.21315939 | 14 |
| 3.3 | qph | 0 | 0 | 1090.903586 | 83.22685319 | 8 |
| 3.3 | qth | 0 | 0.111111111 | 1057.231792 | 49.55505945 | 14 |
| 3.3 | pth | 0 | 0.111111111 | 1055.134758 | 47.45802502 | 13 |
| 3.3 | lqpt | 0 | 0.111111111 | 1064.642674 | 56.96594156 | 15 |
| 3.3 | lqph | 0 | 0 | 1090.855502 | 83.17876978 | 8 |
| 3.3 | lqth | 0 | 0 | 1062.488323 | 54.81159083 | 15 |
| 3.3 | lpth | 0 | 0.111111111 | 1056.663201 | 48.98646812 | 13 |
| 3.3 | lqpth | 0 | 0.111111111 | 1064.610343 | 56.93361073 | 15 |
| 3.4 | l | 0 | 0 | 1139.972699 | 132.2959664 | 6 |
| 3.4 | q | 0 | 0 | 1161.103081 | 153.4263482 | 6 |
| 3.4 | p | 0 | 0 | 1136.378616 | 128.7018831 | 6 |
| 3.4 | t | 0 | 0 | 1042.10276 | 34.42602753 | 10 |
| 3.4 | h | 0 | 0 | 1206.176256 | 198.499524 | 24 |
| 3.4 | lq | 0 | 0 | 1128.147537 | 120.4708046 | 7 |
| 3.4 | lp | 0 | 0 | 1129.726926 | 122.0501936 | 6 |
| 3.4 | lt | 0 | 0.111111111 | 1057.345402 | 49.66866989 | 13 |
| 3.4 | lh | 0 | 0 | 1087.146703 | 79.46997043 | 7 |
| 3.4 | qp | 0 | 0 | 1124.223682 | 116.5469497 | 6 |
| 3.4 | qt | 0 | 0.111111111 | 1054.325325 | 46.64859231 | 12 |
| 3.4 | qh | 0 | 0 | 1092.030663 | 84.35393099 | 7 |
| 3.4 | pt | 0 | 0.111111111 | 1057.665754 | 49.98902185 | 13 |
| 3.4 | ph | 0 | 0 | 1112.902441 | 105.2257084 | 10 |
| 3.4 | th | 0 | 0 | 1056.01954 | 48.34280736 | 14 |
| 3.4 | lqp | 0 | 0 | 1123.79507 | 116.1183377 | 6 |
| 3.4 | lqt | 0 | 0.111111111 | 1057.504442 | 49.82770915 | 13 |
| 3.4 | lqh | 0 | 0 | 1084.051656 | 76.3749239 | 7 |
| 3.4 | lpt | 0 | 0.111111111 | 1059.257402 | 51.58066955 | 13 |
| 3.4 | lph | 0 | 0 | 1093.03634 | 85.35960718 | 8 |
| 3.4 | qpt | 0 | 0.111111111 | 1062.369545 | 54.69281261 | 14 |
| 3.4 | qph | 0 | 0 | 1093.137571 | 85.46083896 | 8 |
| 3.4 | qth | 0 | 0.111111111 | 1059.828795 | 52.15206248 | 14 |
| 3.4 | pth | 0 | 0.111111111 | 1057.646144 | 49.96941124 | 13 |
| 3.4 | lqpt | 0 | 0.111111111 | 1067.142559 | 59.46582682 | 15 |
| 3.4 | lqph | 0 | 0 | 1092.990082 | 85.31334988 | 8 |
| 3.4 | lqth | 0 | 0 | 1069.846152 | 62.1694196 | 16 |
| 3.4 | lpth | 0 | 0.111111111 | 1059.165803 | 51.48907034 | 13 |
| 3.4 | lqpth | 0 | 0.111111111 | 1067.135278 | 59.45854552 | 15 |
| 3.5 | l | 0 | 0 | 1140.623877 | 132.9471446 | 6 |
| 3.5 | q | 0 | 0 | 1162.536486 | 154.8597532 | 6 |
| 3.5 | p | 0 | 0 | 1136.945959 | 129.2692269 | 6 |
| 3.5 | t | 0 | 0 | 1044.375018 | 36.69828514 | 10 |
| 3.5 | h | 0 | 0 | 1201.022789 | 193.3460564 | 23 |
| 3.5 | lq | 0 | 0 | 1129.498559 | 121.821826 | 7 |
| 3.5 | lp | 0 | 0 | 1130.214755 | 122.5380221 | 6 |
| 3.5 | lt | 0 | 0.111111111 | 1059.851365 | 52.17463294 | 13 |
| 3.5 | lh | 0 | 0 | 1089.124344 | 81.44761164 | 7 |
| 3.5 | qp | 0 | 0 | 1125.434164 | 117.7574311 | 6 |
| 3.5 | qt | 0 | 0.111111111 | 1056.663284 | 48.98655188 | 12 |
| 3.5 | qh | 0 | 0 | 1094.22904 | 86.55230708 | 7 |
| 3.5 | pt | 0 | 0.111111111 | 1060.121293 | 52.44456097 | 13 |
| 3.5 | ph | 0 | 0 | 1114.876482 | 107.1997496 | 10 |
| 3.5 | th | 0 | 0 | 1058.408121 | 50.73138804 | 14 |
| 3.5 | lqp | 0 | 0 | 1124.988645 | 117.311913 | 6 |
| 3.5 | lqt | 0 | 0.111111111 | 1060.174753 | 52.49802004 | 13 |
| 3.5 | lqh | 0 | 0 | 1086.098201 | 78.42146804 | 7 |
| 3.5 | lpt | 0 | 0.111111111 | 1057.624362 | 49.9476295 | 12 |
| 3.5 | lph | 0 | 0 | 1095.361369 | 87.68463655 | 8 |
| 3.5 | qpt | 0 | 0.111111111 | 1065.189072 | 57.5123393 | 14 |
| 3.5 | qph | 0 | 0 | 1095.369087 | 87.69235473 | 8 |
| 3.5 | qth | 0 | 0.111111111 | 1062.430741 | 54.75400857 | 14 |
| 3.5 | pth | 0 | 0.111111111 | 1060.14657 | 52.46983719 | 13 |
| 3.5 | lqpt | 0 | 0.111111111 | 1069.977689 | 62.30095631 | 15 |
| 3.5 | lqph | 0 | 0 | 1095.24553 | 87.56879782 | 8 |
| 3.5 | lqth | 0 | 0 | 1072.584353 | 64.90762036 | 16 |
| 3.5 | lpth | 0 | 0.111111111 | 1057.533268 | 49.85653549 | 12 |
| 3.5 | lqpth | 0 | 0.111111111 | 1069.706633 | 62.02990016 | 15 |
| 3.6 | l | 0 | 0 | 1141.298127 | 133.6213943 | 6 |
| 3.6 | q | 0 | 0 | 1163.958809 | 156.2820769 | 6 |
| 3.6 | p | 0 | 0 | 1137.532379 | 129.8556467 | 6 |
| 3.6 | t | 0 | 0 | 1046.712034 | 39.0353013 | 10 |
| 3.6 | h | 0 | 0 | 1204.061104 | 196.3843712 | 23 |
| 3.6 | lq | 0 | 0 | 1130.870657 | 123.1939244 | 7 |
| 3.6 | lp | 0 | 0 | 1130.761837 | 123.0851049 | 6 |
| 3.6 | lt | 0 | 0.111111111 | 1062.362038 | 54.6853056 | 13 |
| 3.6 | lh | 0 | 0 | 1094.104209 | 86.42747615 | 8 |
| 3.6 | qp | 0 | 0 | 1126.598708 | 118.9219753 | 6 |
| 3.6 | qt | 0 | 0.111111111 | 1059.035643 | 51.3589107 | 12 |
| 3.6 | qh | 0 | 0 | 1096.372353 | 88.69562002 | 7 |
| 3.6 | pt | 0 | 0.111111111 | 1062.697112 | 55.02037901 | 13 |
| 3.6 | ph | 0 | 0 | 1120.407074 | 112.7303415 | 11 |
| 3.6 | th | 0 | 0 | 1060.834412 | 53.1576791 | 14 |
| 3.6 | lqp | 0 | 0 | 1126.115411 | 118.4386785 | 6 |
| 3.6 | lqt | 0 | 0.111111111 | 1058.705054 | 51.02832115 | 12 |
| 3.6 | lqh | 0 | 0 | 1091.097345 | 83.42061288 | 8 |
| 3.6 | lpt | 0 | 0.111111111 | 1059.76848 | 52.0917472 | 12 |
| 3.6 | lph | 0 | 0 | 1097.573768 | 89.89703522 | 8 |
| 3.6 | qpt | 0 | 0.111111111 | 1067.69971 | 60.022977 | 14 |
| 3.6 | qph | 0 | 0 | 1097.767937 | 90.09120423 | 8 |
| 3.6 | qth | 0 | 0.111111111 | 1065.032119 | 57.35538648 | 14 |
| 3.6 | pth | 0 | 0.111111111 | 1062.711415 | 55.03468205 | 13 |
| 3.6 | lqpt | 0 | 0.111111111 | 1067.888893 | 60.21216027 | 14 |
| 3.6 | lqph | 0 | 0 | 1097.488506 | 89.81177399 | 8 |
| 3.6 | lqth | 0 | 0 | 1070.291387 | 62.61465482 | 15 |
| 3.6 | lpth | 0 | 0.111111111 | 1059.652269 | 51.97553631 | 12 |
| 3.6 | lqpth | 0 | 0.111111111 | 1072.071919 | 64.39518645 | 15 |
| 3.7 | l | 0 | 0 | 1141.974814 | 134.2980819 | 6 |
| 3.7 | q | 0 | 0 | 1165.36634 | 157.6896075 | 6 |
| 3.7 | p | 0 | 0 | 1138.102455 | 130.4257227 | 6 |
| 3.7 | t | 0 | 0 | 1049.026406 | 41.34967367 | 10 |
| 3.7 | h | 0 | 0 | 1184.677499 | 177.0007666 | 20 |
| 3.7 | lq | 0 | 0 | 1132.203557 | 124.5268242 | 7 |
| 3.7 | lp | 0 | 0 | 1128.511226 | 120.8344939 | 5 |
| 3.7 | lt | 0 | 0.111111111 | 1064.89055 | 57.21381789 | 13 |
| 3.7 | lh | 0 | 0 | 1093.220333 | 85.54360061 | 7 |
| 3.7 | qp | 0 | 0 | 1127.755754 | 120.0790217 | 6 |
| 3.7 | qt | 0 | 0.111111111 | 1061.447245 | 53.7705123 | 12 |
| 3.7 | qh | 0 | 0 | 1098.54975 | 90.87301748 | 7 |
| 3.7 | pt | 0 | 0.111111111 | 1065.233868 | 57.55713593 | 13 |
| 3.7 | ph | 0 | 0 | 1122.50523 | 114.8284972 | 11 |
| 3.7 | th | 0 | 0 | 1063.025513 | 55.34878026 | 14 |
| 3.7 | lqp | 0 | 0 | 1127.201314 | 119.5245819 | 6 |
| 3.7 | lqt | 0 | 0.111111111 | 1061.052906 | 53.3761734 | 12 |
| 3.7 | lqh | 0 | 0 | 1090.143151 | 82.46641815 | 7 |
| 3.7 | lpt | 0 | 0.111111111 | 1062.006856 | 54.33012372 | 12 |
| 3.7 | lph | 0 | 0 | 1096.87961 | 89.20287706 | 7 |
| 3.7 | qpt | 0 | 0.111111111 | 1065.932447 | 58.25571497 | 13 |
| 3.7 | qph | 0 | 0 | 1096.981777 | 89.30504474 | 7 |
| 3.7 | qth | 0 | 0.111111111 | 1063.431765 | 55.75503224 | 13 |
| 3.7 | pth | 0 | 0.111111111 | 1065.235452 | 57.55871904 | 13 |
| 3.7 | lqpt | 0 | 0.111111111 | 1070.265548 | 62.58881584 | 14 |
| 3.7 | lqph | 0 | 0 | 1096.975867 | 89.29913406 | 7 |
| 3.7 | lqth | 0 | 0 | 1072.799245 | 65.12251276 | 15 |
| 3.7 | lpth | 0 | 0.111111111 | 1061.922252 | 54.24551962 | 12 |
| 3.7 | lqpth | 0 | 0.111111111 | 1070.187252 | 62.51051923 | 14 |
| 3.8 | l | 0 | 0 | 1142.650898 | 134.9741656 | 6 |
| 3.8 | q | 0 | 0 | 1166.770971 | 159.0942382 | 6 |
| 3.8 | p | 0 | 0 | 1138.666803 | 130.9900708 | 6 |
| 3.8 | t | 0 | 0 | 1051.413874 | 43.73714176 | 10 |
| 3.8 | h | 0 | 0 | 1202.399926 | 194.7231935 | 22 |
| 3.8 | lq | 0 | 0 | 1133.518068 | 125.8413353 | 7 |
| 3.8 | lp | 0 | 0 | 1128.914459 | 121.2377264 | 5 |
| 3.8 | lt | 0 | 0.111111111 | 1063.372733 | 55.69600089 | 12 |
| 3.8 | lh | 0 | 0 | 1095.207614 | 87.53088112 | 7 |
| 3.8 | qp | 0 | 0 | 1128.745845 | 121.0691129 | 6 |
| 3.8 | qt | 0 | 0.111111111 | 1060.009341 | 52.33260871 | 11 |
| 3.8 | qh | 0 | 0 | 1100.688854 | 93.01212169 | 7 |
| 3.8 | pt | 0 | 0.111111111 | 1067.819387 | 60.14265444 | 13 |
| 3.8 | ph | 0 | 0 | 1120.915673 | 113.2389407 | 10 |
| 3.8 | th | 0 | 0 | 1061.01139 | 53.33465712 | 13 |
| 3.8 | lqp | 0 | 0 | 1128.238047 | 120.5613143 | 6 |
| 3.8 | lqt | 0 | 0 | 1067.416358 | 59.73962546 | 13 |
| 3.8 | lqh | 0 | 0 | 1095.06579 | 87.38905748 | 8 |
| 3.8 | lpt | 0 | 0.111111111 | 1064.194918 | 56.51818563 | 12 |
| 3.8 | lph | 0 | 0 | 1098.967955 | 91.29122248 | 7 |
| 3.8 | qpt | 0 | 0.111111111 | 1068.180746 | 60.50401309 | 13 |
| 3.8 | qph | 0 | 0 | 1099.024033 | 91.34730005 | 7 |
| 3.8 | qth | 0 | 0.111111111 | 1065.87974 | 58.20300785 | 13 |
| 3.8 | pth | 0 | 0.111111111 | 1067.907444 | 60.2307112 | 13 |
| 3.8 | lqpt | 0 | 0.111111111 | 1072.307814 | 64.63108191 | 14 |
| 3.8 | lqph | 0 | 0 | 1098.968958 | 91.29222512 | 7 |
| 3.8 | lqth | 0 | 0 | 1075.255774 | 67.57904119 | 15 |
| 3.8 | lpth | 0 | 0.111111111 | 1064.162076 | 56.48534383 | 12 |
| 3.8 | lqpth | 0 | 0.111111111 | 1072.389632 | 64.71289924 | 14 |
| 3.9 | l | 0 | 0 | 1143.336497 | 135.6597649 | 6 |
| 3.9 | q | 0 | 0 | 1168.166985 | 160.4902525 | 6 |
| 3.9 | p | 0 | 0 | 1139.246319 | 131.5695869 | 6 |
| 3.9 | t | 0 | 0 | 1050.306776 | 42.63004347 | 9 |
| 3.9 | h | 0 | 0 | 1205.737465 | 198.0607327 | 22 |
| 3.9 | lq | 0 | 0 | 1134.771404 | 127.0946714 | 7 |
| 3.9 | lp | 0 | 0 | 1129.314218 | 121.6374858 | 5 |
| 3.9 | lt | 0 | 0.111111111 | 1065.815031 | 58.13829897 | 12 |
| 3.9 | lh | 0 | 0 | 1097.212918 | 89.53618593 | 7 |
| 3.9 | qp | 0 | 0 | 1129.737909 | 122.0611765 | 6 |
| 3.9 | qt | 0 | 0.111111111 | 1062.299632 | 54.62289998 | 11 |
| 3.9 | qh | 0 | 0 | 1102.790859 | 95.11412695 | 7 |
| 3.9 | pt | 0 | 0.111111111 | 1070.429872 | 62.75313943 | 13 |
| 3.9 | ph | 0 | 0 | 1119.606772 | 111.9300395 | 9 |
| 3.9 | th | 0 | 0 | 1063.122752 | 55.44601953 | 13 |
| 3.9 | lqp | 0 | 0 | 1129.201938 | 121.5252056 | 6 |
| 3.9 | lqt | 0 | 0 | 1065.898691 | 58.22195836 | 12 |
| 3.9 | lqh | 0 | 0 | 1097.052876 | 89.37614371 | 8 |
| 3.9 | lpt | 0 | 0.111111111 | 1066.468027 | 58.79129482 | 12 |
| 3.9 | lph | 0 | 0 | 1101.129557 | 93.4528241 | 7 |
| 3.9 | qpt | 0 | 0.111111111 | 1070.359181 | 62.68244856 | 13 |
| 3.9 | qph | 0 | 0 | 1101.319939 | 93.64320687 | 7 |
| 3.9 | qth | 0 | 0.111111111 | 1068.467183 | 60.79045049 | 13 |
| 3.9 | pth | 0 | 0.111111111 | 1066.552764 | 58.87603113 | 12 |
| 3.9 | lqpt | 0 | 0.111111111 | 1066.451908 | 58.77517567 | 12 |
| 3.9 | lqph | 0 | 0 | 1101.027194 | 93.35046111 | 7 |
| 3.9 | lqth | 0 | 0 | 1082.74101 | 75.06427746 | 16 |
| 3.9 | lpth | 0 | 0.111111111 | 1066.30994 | 58.63320731 | 12 |
| 3.9 | lqpth | 0 | 0 | 1074.529442 | 66.85270922 | 14 |
| 4 | l | 0 | 0 | 1144.028508 | 136.351776 | 6 |
| 4.0 | q | 0 | 0 | 1169.548707 | 161.8719747 | 6 |
| 4.0 | p | 0 | 0 | 1139.83884 | 132.1621076 | 6 |
| 4.0 | t | 0 | 0 | 1052.395771 | 44.7190382 | 9 |
| 4.0 | h | 0 | 0 | 1176.871974 | 169.1952414 | 17 |
| 4.0 | lq | 0 | 0 | 1136.017847 | 128.3411149 | 7 |
| 4.0 | lp | 0 | 0 | 1129.725509 | 122.0487761 | 5 |
| 4.0 | lt | 0 | 0 | 1068.225886 | 60.54915325 | 12 |
| 4.0 | lh | 0 | 0 | 1102.130614 | 94.45388125 | 8 |
| 4.0 | qp | 0 | 0 | 1130.704786 | 123.0280533 | 6 |
| 4.0 | qt | 0 | 0.111111111 | 1064.613822 | 56.93708955 | 11 |
| 4.0 | qh | 0 | 0 | 1104.973335 | 97.29660234 | 7 |
| 4.0 | pt | 0 | 0 | 1065.172788 | 57.4960555 | 11 |
| 4.0 | ph | 0 | 0 | 1124.798494 | 117.1217616 | 10 |
| 4.0 | th | 0 | 0 | 1069.492119 | 61.8153865 | 14 |
| 4.0 | lqp | 0 | 0 | 1130.135716 | 122.4589837 | 6 |
| 4.0 | lqt | 0 | 0 | 1068.417965 | 60.7412328 | 12 |
| 4.0 | lqh | 0 | 0 | 1096.095667 | 88.41893423 | 7 |
| 4.0 | lpt | 0 | 0.111111111 | 1068.696759 | 61.02002698 | 12 |
| 4.0 | lph | 0 | 0 | 1106.124126 | 98.44739346 | 8 |
| 4.0 | qpt | 0 | 0 | 1068.53297 | 60.8562377 | 12 |
| 4.0 | qph | 0 | 0 | 1103.371492 | 95.69475983 | 7 |
| 4.0 | qth | 0 | 0.111111111 | 1070.964258 | 63.28752592 | 13 |
| 4.0 | pth | 0 | 0 | 1065.216558 | 57.53982518 | 11 |
| 4.0 | lqpt | 0 | 0 | 1068.465632 | 60.78889985 | 12 |
| 4.0 | lqph | 0 | 0 | 1103.136773 | 95.46004031 | 7 |
| 4.0 | lqth | 0 | 0 | 1076.045894 | 68.36916172 | 14 |
| 4.0 | lpth | 0 | 0.111111111 | 1068.612949 | 60.93621604 | 12 |
| 4.0 | lqpth | 0 | 0 | 1068.445635 | 60.76890297 | 12 |

Note: RM is Regularization multiplier, FC is Feature class, L is linear, Q is quadratic, P is product, T is threshold, and H is hinge.

**Table S4 CBI, AUC and TSS index during 10 repetitions of the modeling procedure**

| **Parameters** | **Rep. 1** | **Rep. 2** | **Rep. 3** | **Rep. 4** | **Rep. 5** | **Rep. 6** | **Rep. 7** | **Rep. 8** | **Rep. 9** | **Rep. 10** | **Avg.** | **SD** |
| --- | --- | --- | --- | --- | --- | --- | --- | --- | --- | --- | --- | --- |
| **CBI** | 0.887 | 0.891 | 0.890 | 0.893 | 0.889 | 0.894 | 0.887 | 0.888 | 0.890 | 0.889 | 0.890 | 0.002 |
| **AUC** | 0.963 | 0.963 | 0.965 | 0.965 | 0.965 | 0.962 | 0.964 | 0.962 | 0.961 | 0.963 | 0.9632 | 0.001 |
| **TSS** | 0.818 | 0.819 | 0.821 | 0.822 | 0.822 | 0.820 | 0.821 | 0.819 | 0.819 | 0.820 | 0.820 | 0.001 |

**Table S5 Statistical analysis of environmental variables of *Alligator sinensis*. Min. is minimum, Max. is maximum, OR is optimum range.**

| **Variables** | **Min** | **Max** | **Mean** | **OR** |
| --- | --- | --- | --- | --- |
| Bio3 | 16.04 | 40.62 | 27.47 | 21.59 ~ 24.49 |
| Bio5 | 11.40 | 34.60 | 30.34 | 32.17 ~ 33.08 |
| Bio6 | -11.1 | 8.3 | 0.33 | -0.55 ~ -0.46 |
| Bio12 | 502.00 | 4264.00 | 1242.43 | 1206.20 ~ 1311.43 |
| Bio15 | 19.13 | 107.50 | 63.94 | 46.35 ~ 49.68 |
| Bio18 | 236.0 | 1754.00 | 495.44 | 495.13 ~ 519.82 |

**Table S6 Barycenter coordinates of** ***Alligator sinensis* under current and future scenarios, considering two Shared Socioeconomic Pathways (SSP126 and SSP585) for the years 2050 and 2070.**

| **Year** | **SSPs** | **Longitude** | **Latitude** |
| --- | --- | --- | --- |
| **Current** | **-** | 1303147.163 | 3426620.198 |
| **2050** | **SSP126** | 1257700.573 | 3394665.105 |
|  | **SSP585** | 1253018.474 | 3438656.718 |
| **2070** | **SSP126** | 1147155.96 | 3370692.546 |
|  | **SSP585** | 1161138.532 | 3378530.378 |

**Table S7 Statistics of center of gravity migration distance of *Alligator sinensis* with two Shared Socioeconomic Pathways (SSP126 and SSP585)**

| **SSPs** | **Years** | **Distance(Km)** |
| --- | --- | --- |
| **126** | **Current→2050s** | 55.5565 |
|  | **Current→2070s** | 165.7141 |
|  | **2050s→2070s** | 44.2401 |
| **585** | **Current→2050s** | 51.5535 |
|  | **Current→2070s** | 149.9303 |
|  | **2050s→2070s** | 109.8048 |

**.**

**Table S8 Superposition results of priority protected areas**

| **Value** | **Count** | **Area (Km^2^)** |
| --- | --- | --- |
| 0 | 1,872,844 | 1,870,601.74 |
| 1 | 8,732 | 8706.17 |
| 2 | 388 | 383.68 |
| 3 | 4 | 4.00 |
| 4 | 0 | 0 |
| 5 | 0 | 0 |


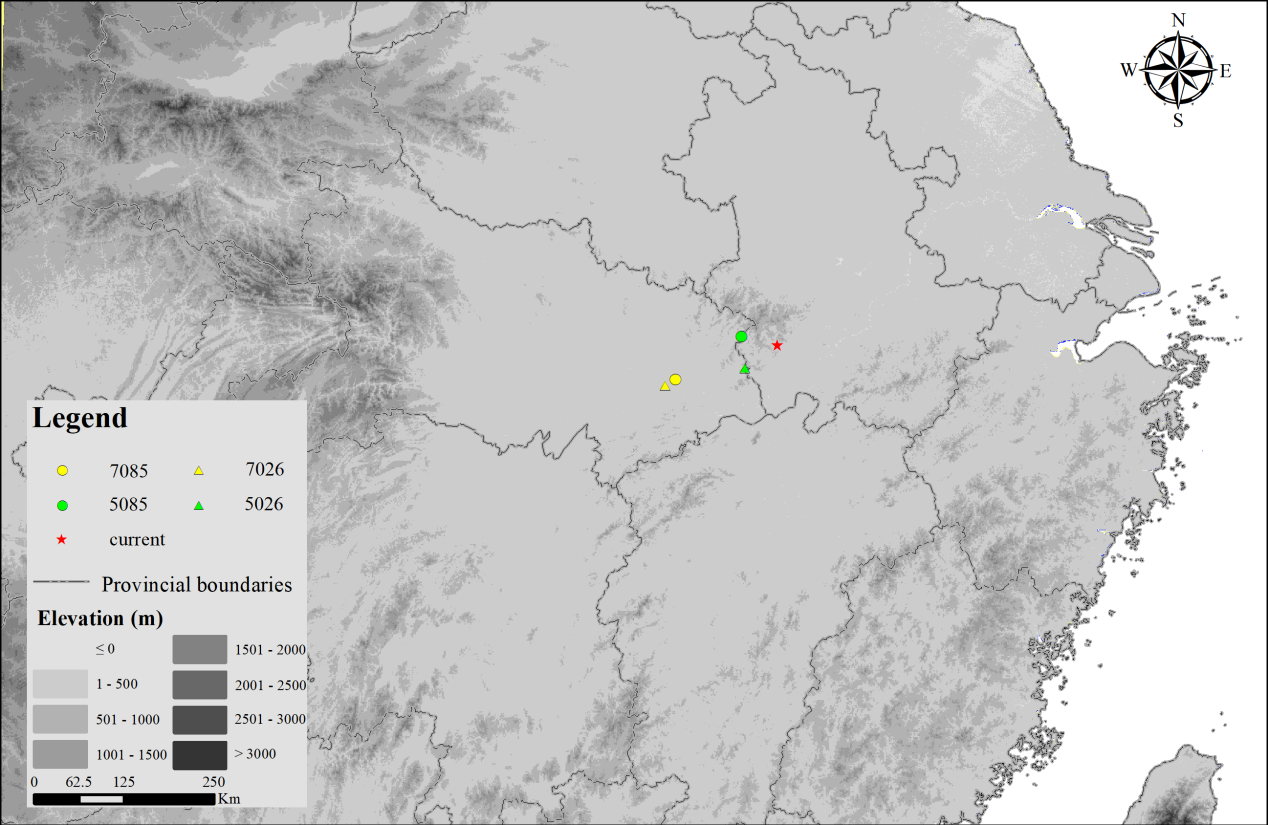


**Fig. S1 Barycenter transfer of *Alligator sinensis* in China.**

**
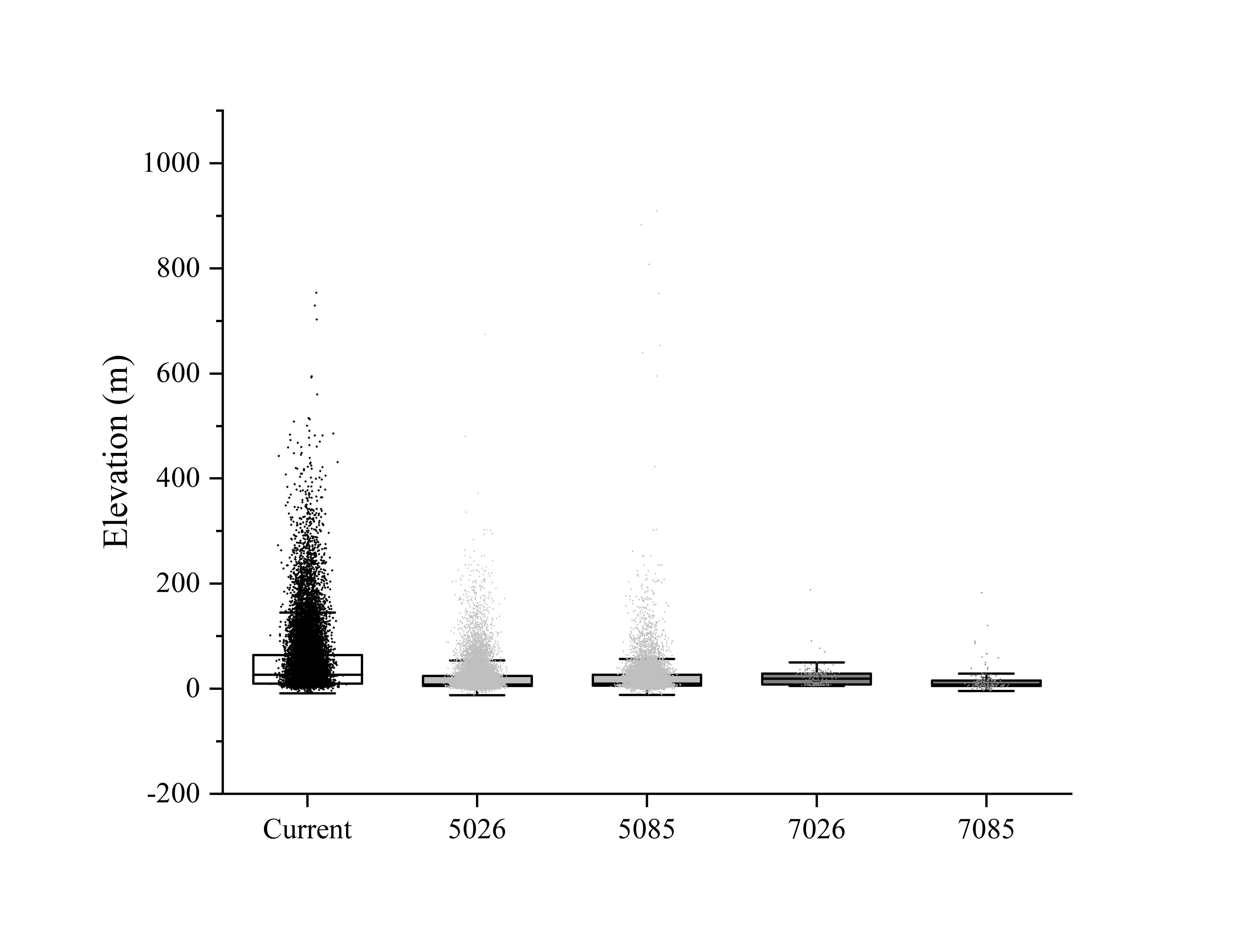
**

**Fig. S2 Altitudinal distribution of the Chinese alligator under five climatic scenarios**

**
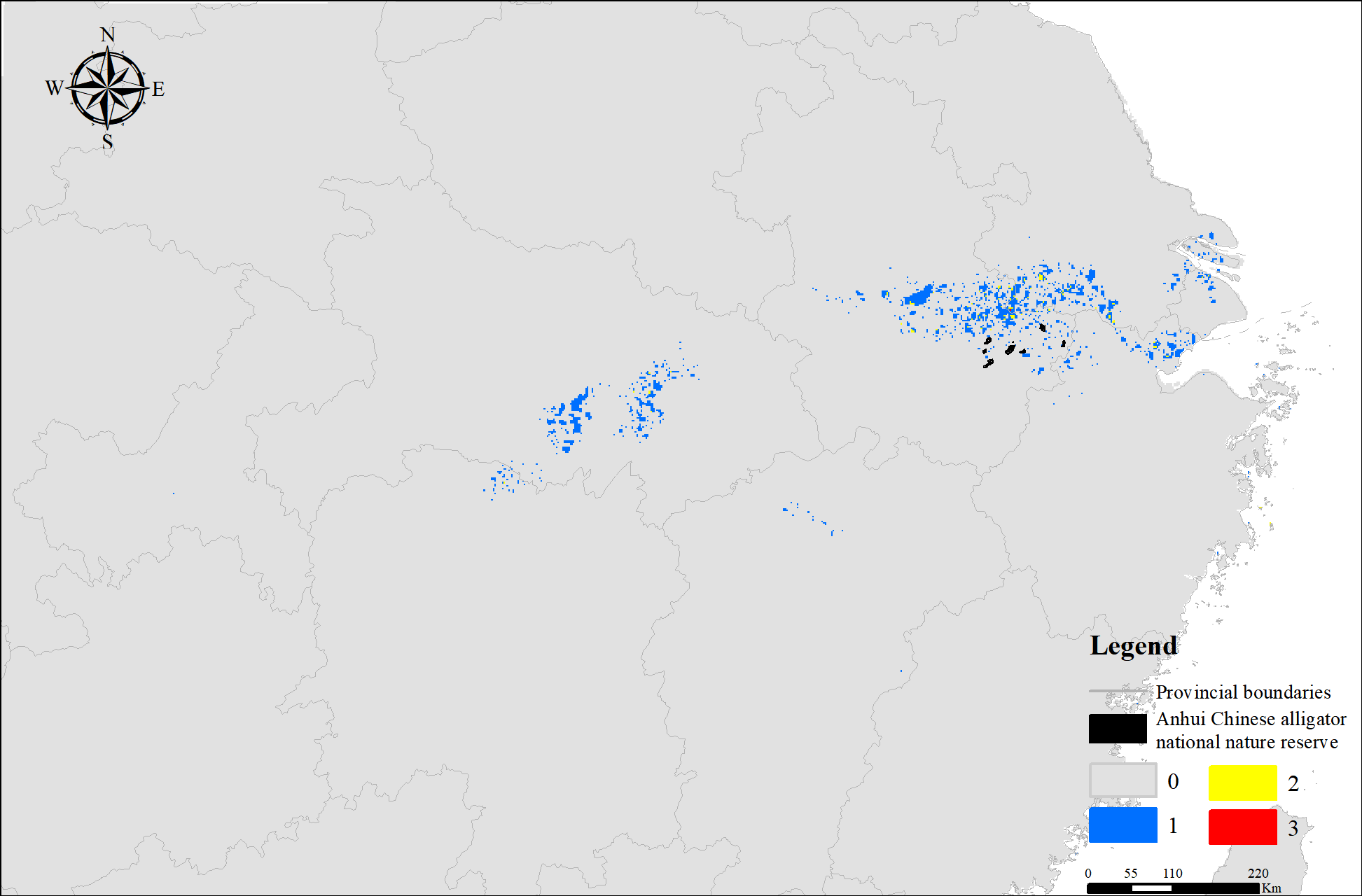
**

**Fig. S3 Superimposed map of priority protection areas for Chinese alligators under 5 climate scenarios.**

**
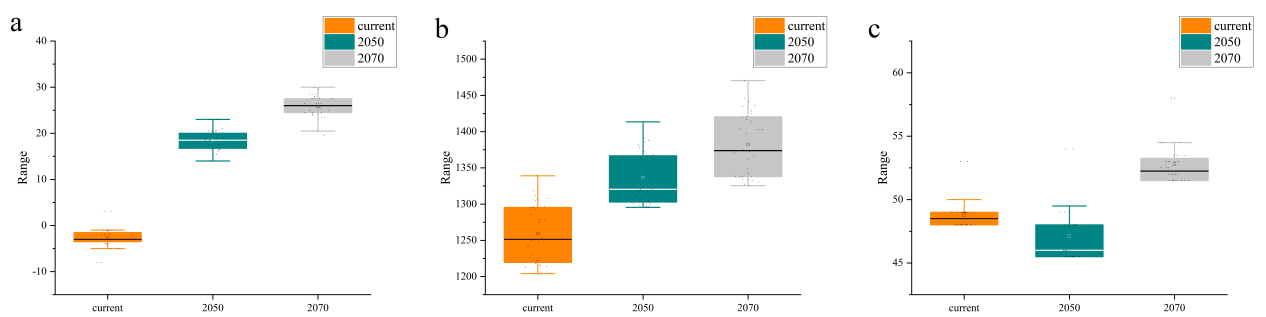
**

**Fig.S4 Future climate change with habitat of *Alligator sinensis* (a: Minimum temperature of coldest month; b: Annual precipitation; c: Precipitation seasonality).**
